# Co-precipitation Synthesis of Near-infrared Iron Oxide Nanocrystals on Magnetically Targeted Imaging and Photothermal Cancer Therapy via Photoablative Protein Denature

**DOI:** 10.7150/ntno.24124

**Published:** 2019-05-25

**Authors:** Wei-Jhe Syu, Chih-Chia Huang, Jong-Kai Hsiao, Yao-Chang Lee, Yu-Tsang Huang, Parthiban Venkatesan, Ping-Shan Lai

**Affiliations:** 1 Department of Chemistry, National Chung Hsing University, Taichung City 402, Taiwan; 2 Department of Photonics, National Cheng Kung University, Tainan City 701, Taiwan; 3 Department of Medical Imaging, Buddhist Tzu Chi General Hospital, Taipei Branch, New Taipei City 231, Taiwan; 4 National Synchrotron Radiation Research Center, Hsinchu Science Park, Hsinchu 30076, Taiwan; 5 Menglinbeier Clinic, Taichung City 407, Taiwan

**Keywords:** photothermal therapy, Fe_3_O_4_ nanocrystals, magnetic field targeting, near-infrared

## Abstract

Near-infrared (NIR)-based nanomaterials that provide efficient tumor ablation for cancer therapy have been reported. However, the issues of biocompatibility of metals or ions in inorganic nanoparticles systems such as copper and gold nanoparticles are still a matter of concern. In this study, we developed a facile and ligand-assisted co-precipitation method to synthesize biocompatible iron oxide (IO) nanocrystals with NIR absorption that provided T2-weighted magnetic resonance (MR) images and photothermal ablation characteristics suitable for cancer theranostics. Our results showed that 150-nm particles can be synthesized and optimized by using different amounts of ligand. NIR-IO nanocrystals of this size showed high photothermal conversion efficiency (21.2%) and T2-weighted MR contrast (transverse relaxivity value approximately 141 S-1 mM-1). The NIR-IO nanocrystals showed no cytotoxicity in HT-29 colorectal cancer cells without irradiation, whereas the viability of cells that received NIR-IO nanocrystals decreased significantly after 808-nm laser irradiation. The mechanism of cell death may involve alterations in protein secondary structure and membrane permeability. For *in vivo* studies, 4-fold enhanced tumor accumulation was significantly observed of NIR-IO nanocrystals with a magnetic field (MF) application, resulting in a 3-fold higher T2-weighted MR signal than that produced by a commercial T2-weighted MR contrast agent (Resovist®) and excellent photothermal efficacy (approximately 53 °C) for cancer treatment. The innovative NIR-IO nanocrystals showed excellent biocompatibility and have great potential as a theranostic agent against cancer.

## 1. Introduction

Enhanced imaging-guided therapy^1^, ^2^ combined with non-invasive treatment to efficiently seek and shrink or cure various types of diseased tissues is highly desired. The theranostic systems for cancer therapy were developed for real time evaluation and optimization of treatment conditions in the period time of treatment and then emerging field toward personalized treatment for the benefit of cancer patients.^3^-^5^ Combined with nanoparticles in minimally invasive treatments and good permeability and retention (EPR) effect, many groups reported nano-hybrid systems^6^, ^7^ to approach image-guided therapies against troublesome cancers such as brain tumors has become a goal of clinical trials based on the unique physicochemical treatments of near-infrared (NIR)-absorption, AC radiofrequency activation, and drug delivery. However, the development of single component nanoparticles for using thermostats remained meet a challenge in the materials synthesis by integrating optical and magnetic features. Although gold-based biomaterials are successful cases for imaging integrated phototherapy 5, 8 e.g., computed tomography-photothermal therapy (CT-PTT) 9, dark-field imaging-PTT 10, surface enhanced Raman scattering-PTT 11, fluorescence-PTT 12, and multiphoton-photothermal therapy (multiphoton-PTT), 13 these gold-based biomaterials required multi-step postsynthesis processes, and cannot be used in non-biological targeting with lower cost such as magnetic field targeting due to lack of magnetic property.

Magnetic iron oxide (IO) nanoparticles have attracted great attention in biomedical applications, such as targeted drug delivery, magnetic resonance imaging (MRI), hyperthermia, and biosensing.^14^-^16^ Our group and many pioneering studies have not only demonstrated the suitability of IO nanoparticles as potential *T_2_* contrast agents due to their superparamagnetic behavior and high biocompatibility 17, but also as a basis for magnetic targeting to enhance the accumulation of clinical drugs 18. In contrast to passive delivery through the enhanced permeability and retention (EPR) of some nanoparticles, which produces a low efficiency of accumulation of therapeutic agents in many cases due to the pathophysiological heterogeneity of tumors 19. Magnetic nanocomposites combined with external magnetic fields (MFs) 20, 21, provides an advantageous strategy for the active targeting of tumors to overcome the insufficient differences in receptor expression levels among patients for receptor expression between normal and cancerous tissues 18, 20 Li et al. used this MF strategy to enhance the accumulation of magnetic nanoparticles on the side of a tumor by 3.5-fold compared with that for a method that used the EPR effect^21^. Zhang et al. also reported that the MF strategy could reduce the effective dosage of chemotherapeutics by half 22, 23. However, the strategy of MF targeting integrated MRI-photothermal system was rarely reported.

To concern the green mass-production and chemical waste, the IO nanoparticles via aqueous synthesis were exhibited excellent results in recent researches. However, theses aqueous synthesis methods had exist some problems such as size distribution widely or water dispersion poorly due to the two important processs: nucleation and crystalline growth were not being controlled easily 24, 25. In recent years, the studies of IO nanocarriers had be well studied, however the studies of these IO nanocarriers with photothermal abilty were relative rarely in the area of IO nanocarriers development. Herein, a modified co-precipitation synthesis of IO nanoparticle with high photothermal efficiency was developed by all-in-one reaction of the very low toxic reactants: FeSO_4_-citrate complex with reductant-NaOH/TMA solution. It was also demonstrated that ligands not only plays an important role to influence composition and size of nanoparticles depends on reduction/oxidation of ligands to Fe^+2^ but also control optical properties for the synthesis of NIR IO nanoparticles via partial oxidation co-precipitate approach. In Our results show that the optical absorption features and crystallinity of the particles in the NIR region (λ = 650-900 nm) increase with the use of smaller amounts of the reaction ligand (i.e., the 808-nm and 660-nm molar extinction coefficients (ε values) of the lower citrate experimental group were approximate 9-fold higher than those of the higher citrate experimental group). As expected, the photothermal evaluation results also showed high ε values for the IO nanocrystals, indicating that they undergo more extensive and more rapid increases in temperature under the same irradiation conditions. Zhou et al. designed Fe/Fe_3_O_4_ nanoparticles required multiplex modulation on the hydrophobic and hydrophilic surface to achieve to target HeLa tumors, and their experimental results showed excellent photo-stimulated therapeutic efficacy.^26^ However, these nanoparticles were synthesized in organic media, an issue of concern for clinical application because a surfactant should be allowable in the preparation process to convert the hydrophobicity of nanoparticles to hydrophilicity. Liao et al. introduced a method of ligand-assisted synthesis in which carboxylate-based ligands were used to increase the interactions between ligand and iron ions to create IO nanostructures with NIR absorption 27, 28. However, the particle size obtained using this method was approximately 300-400 nm, which is too large for passive tumor targeting. Several demonstrations are convincing scientific evidence of the photothermal ablation (PTA) for cancer therapy,^27^, ^29^-^39^ but the studies of efficacy of the magnetic targeting process for improving the PTA and MR imaging of tumor cells during pre-clinical examinations are still less. Herein, to provide a proof-of-concept for the use of MF targeting of tumor sites to greatly enhance PTT efficacy and MR *T_2_* contrast imaging in this study, mice bearing HT-29 tumors were intravenously injected with NIR-IO nanocrystals and biodistribution, *in vitro* and *in vivo* photothermal therapy and MR imaging were evaluated for MF-enhanced cancer theranostics.

## 2. Results and Discussion

### 2.1 Synthesis and Characterization of NIR-Activated IO Nanocrystals

**Figure 1:**
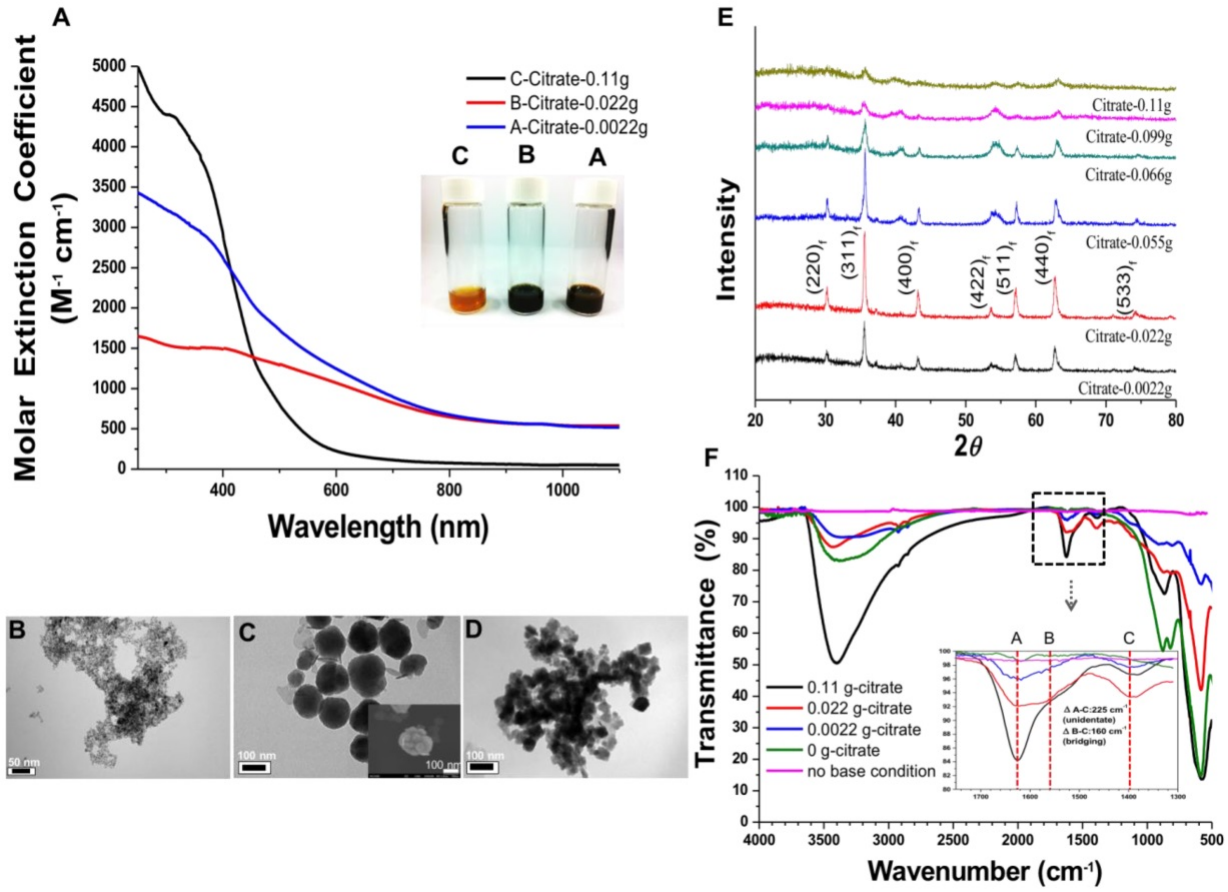
A) Molar extinction coefficient - wavelength diagram and optical image (insert) of NIR-IO nanocrystals. B-D) TEM images of NIR-IO nanocrystals synthesized with 0.11 g, 0.022 g and 0.0022 g of citrate. The scale bar of 1B is 50 nm, 1C and 1D are 100 nm. E) XRD pattern of NIR-IO nanocrystals synthesized using different amounts of citrate assigned to JCPDS file No. 19-0629. F) FTIR measurement of NIR-IO nanocrystals synthesized using different amounts of citrate.

The NIR-IO nanocrystals were synthesized by using 0.11 g of trisodium citrate (citrate), hydrazine monohydrate (N_2_H_4_), and trimesic acid (TMA) as a capping and precipitating agent to react with FeSO_4_ to produce nanocrystals. In this synthesis procedure, the TMA was acted a gentle oxidant to provide ferrous ions and N_2_H_4_ was acted reducing agent, and finally these agents were removed via dialysis. The absorption spectrum (Figure S1) shows that the NIR-IO nanocrystals absorb light extended to long wavelengths including visible (660 nm) and NIR (808 nm) regions. Band around 660 and 808 nm are commonly used in PTT and photodynamic therapy (PDT) 21, 26, 40. To further evaluate the optical properties of the NIR-IO, the extinction coefficient *ε* was determined, as listed in Table 1. The curves of *ε* values-wavelength for NIR-IO prepared with 0.11 g of citrate are presented in Figure 1A, showing 142.49 and 75.63 M^-1^ cm^-1^ at 660 nm and 808 nm, respectively. These *ε* values were higher than those of single-walled carbon nanotubes (CNTs) 41. The transmission electron microscopy (TEM) results showed small (≈4 nm) and uniform nanocrystals (Figure 1B). Previous research has shown that citrate influences the IO particle size by inhibiting the nucleation process and crystal growth 25, 42. Therefore, when high amounts citrates were added to the reaction, smaller nanocrystals were formed via the co-precipitation method.

In addition, the utilization of different concentrations of citrate modified the optical properties of IO citrate into the red-NIR wavelength region (Figure S1). Aqueous NIR-IO solution color was visualized as dark brown, black and orange at 0.0022 g (A sample), 0.022 g (B sample) and 0.11 g (C sample) of citrate groups, respectively (Figure 1A insert). The *ε* values in the plot of *ε* value versus wavelength for NIR-IO increased with decreasing citrate from 0.022 g to 0.0022 g and increasing citrate to 0.11 g in the UV regions (250 to 400 nm). Moreover, the higher *ε* value at a wavelength of 600-808 nm was observed for decreasing citrate from 0.11 g to 0.022 g (Figure 1A). The NIR-IO nanocrystals displayed high *ε* values at a wavelength of 808 nm and approaching to 650 M^-1^ cm^-1^, which can be controlled by varying citrate concentration. Even though the *ε* value of the NIR-IO nanocrystals at 660 nm (almost 950 M^-1^ cm^-1^) was higher than that at 808 nm, the longer irradiation wavelength of 808 nm has been preferred for cancer therapy due to its stronger tissue penetration. The optical properties of NIR-IO-A (citrate-0.0022 g) group and NIR-IO-B (citrate-0.022 g) group are very similar (~950 nm) in the wavelength of 808 nm. However, the experimental group of citrate at 0.0022 g has prone to more aggregation (Figure 1D) owing to the lower availability of citrate to stabilize iron oxide nanoparticles and properties of nanomaterials potential in cancer therapy PTT application.

Intriguingly, the influence of citrate on NIR-IO nanocrystals may result from its effects on nucleation and crystal growth. In a study within the framework of classical nucleation theory, Baumgartner et al. demonstrated the pathway of the primary particles from the bulk phase 43. They estimated the energy barriers for the nucleation of the amorphous and crystalline phases and proposed that the synthesis of magnetite via co-precipitation occurred by the accretion of clusters/primary particles to form a bulk crystalline phase. In our experiments, we also found that the reflection peak of NIR-IO became sharper with lower values of the citrate concentration during synthesis (Figure 1E). The crystallinity of the NIR-IO nanocrystals decreased when the amount of citrate was increased, suggesting the formation of small IO nanocrystals, consistent with the TEM observations. The large amounts of citrate occupying the surface of the primary particles may have prevented these primary particles from undergoing crystal growth (Figure 1E). The FT-IR result showed absorption at 1630 cm^-1^, also indicating that more citrate was chelated with the NIR-IO nanocrystals when larger amounts of citrate were used (Figure 1F). These results indicate that citrate can regulate IO crystallization. However, as shown in the TEM images of NIR-IO in Figure 1D, particles of larger size (≥50 nm) were found when less citrate was added, and large numbers of small-size particles appeared when the amount of citrate was increased.

**Figure 2:**
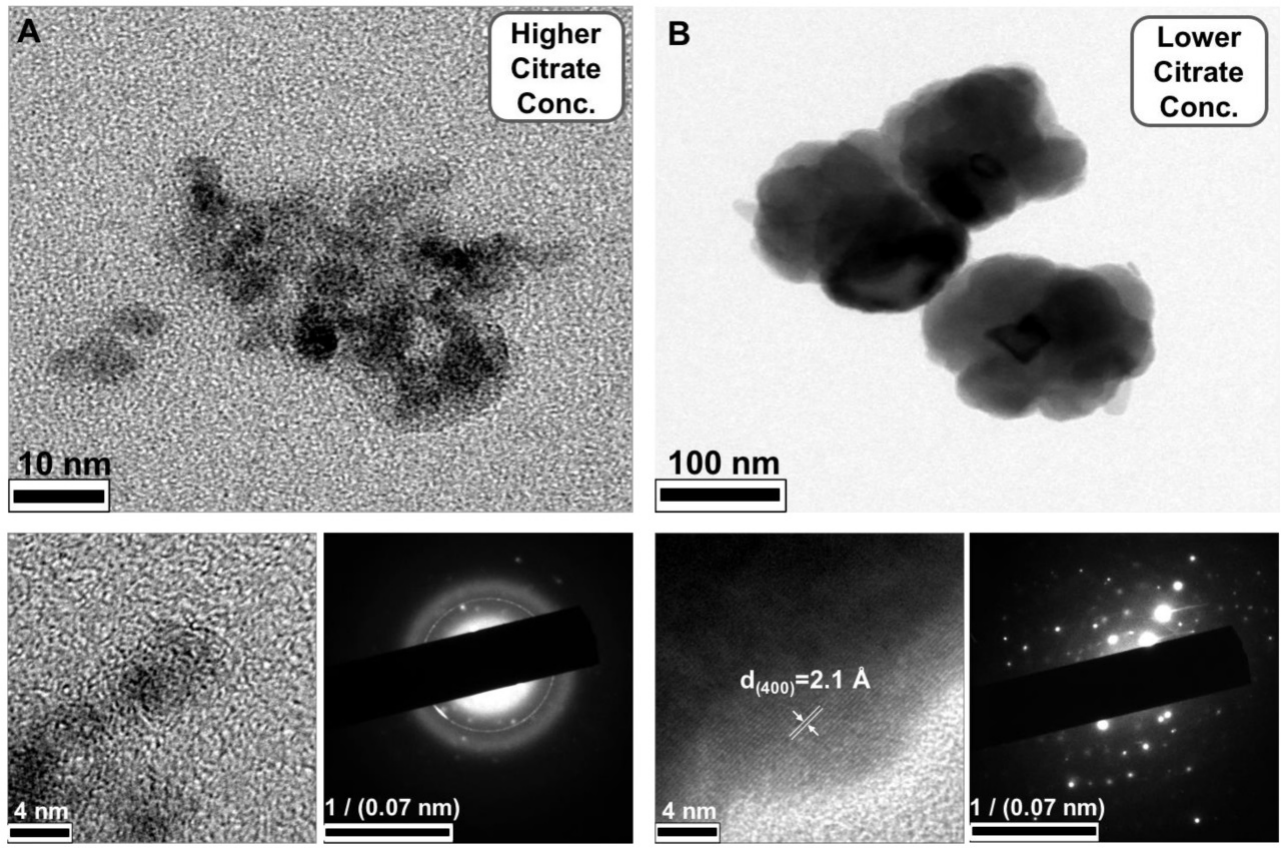
HR-TEM images and ED pattern of NIR-IO nanocrystals obtained via A) high citrate weight (0.11 g) and B) low citrate weight (0.022 g) synthesis.

In addition to showing the influence of citrate on particle size, our results demonstrated the phenomenon of a blueshift in absorption with decreasing particle size. Barnakov and co-workers also used the Faraday rotation spectrum to show similar results concerning the relationship between particle size and optical properties.^44^ This change in the optical properties of particles with decreasing size could be due to a decrease in the number of equivalence sites or an increase in the distance between cations and resulting alterations in the intervalence charge transfer (IVCT) 44, 45. To further investigate the structure of the NIR-IO nanocrystals, high-resolution TEM (HR-TEM) was performed. As shown in Figure 2, particles of small size, irregular shape, and poor crystalline structure were formed when 0.11 g of sodium citrate was used, whereas nanocrystals with approximately 150 nm (Figure 1C) and an interplanar distance of 2.1 Å for the (400) planes of fcc-structures Fe_3_O_4_ were obtained when the amount of sodium citrate was decreased to 0.022 g. The aggregation of smaller particles into larger particles (Figure 1C, image insert) was also observed by SEM. Large-scale analysis was also performed using ED measurement, which showed ring- and dot-patterns for the syntheses with sodium citrate at high and low concentrations, respectively (Figure 2). The fast Fourier transform (FFT) pattern of HR-TEM images also provided evidence about crystalline transformation from interplanar distance of 2.9 Å for the (220) planes and 2.5 Å for the (311) planes to amorphous particles via controlling citrate amounts from 0.022g to 0.11g (Figure S2).

The relationship of ligand to IO nanocrystals was also investigated because ligand chelation influences the optical properties of IO nanocrystals. The FT-IR spectra of NIR-IO nanocrystals prepared with different amounts of citrate were measured (Figure 1F). The absorption bands at 1635 cm^-1^ (A), 1540 cm^-1^ (B), and 1405 cm^-1^ (C) are ascribed to carboxylate groups, whereas the band at 570 cm^-1^ is caused by Fe-O vibrations in Fe_3_O_4_ structure; there, A and B correspond to asymmetrical vibrations of carboxylate (ν_as_(COO)^-^), and C corresponds to the symmetrical vibration of carboxylate (ν_s_(COO)^-^) 46. The information obtained from the spectra confirmed that citrate is coordinated with the NIR-IO nanocrystals. Other studies have demonstrated that ligands such as citrate and trimesic acid can enhance surface effects and produce a larger transition probability of d-d features for the Fe^+2^ and Fe^+3^ ions in the visible-NIR wavelength range 27 Sadat et. al. had reported the detailed electronic bands structure via photoluminescence experiment. The results showed energy of 3.04 eV on Fe_3_O_4_ was attributed via electron transfer from the valence band (O (2*_p_*) to crystal field (*e_g_*)), 2.2 eV was attributed via electron transition from *t_2g_* to *e_g_* (octahedral site), and 0.9 eV was attributed via electron transition from *e* to *t_2_* (tetrahedral site). Sadat et. al. also reported Fe_3_O_4_ nanocrystals possess possible of energy levels, ~ 840 nm (1.47 eV) by the electron traps on the tetrahedral site, that are associated with the oxygen vacancies 47. Furthermore, some studies also provided other explanation that NIR-IO nanocrystals possess two possible types of energy levels, ≈1200 nm by Fe^+2^ ions and 600-900 nm by ^6^A_1_ ↔ ^4^T_1_ + ^4^T_2_ of Fe^+3^ ions 45, 48.

**Figure 3:**
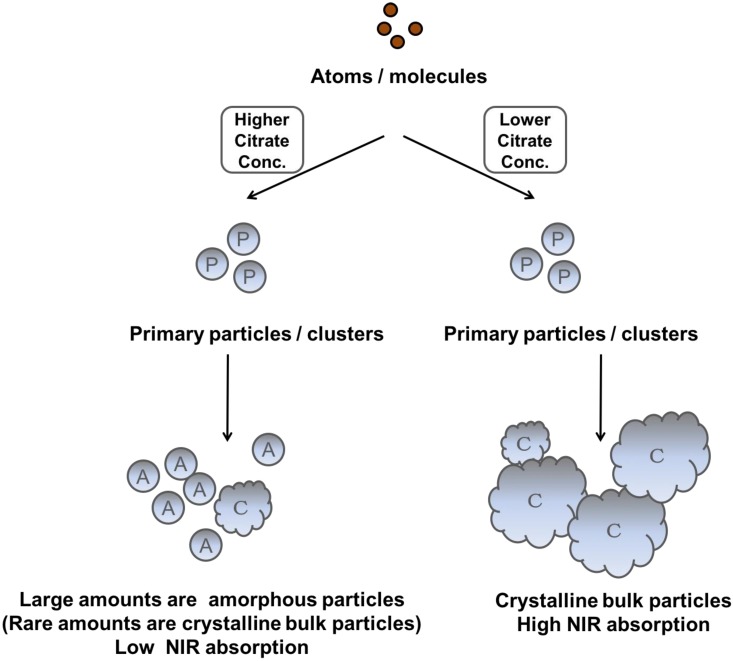
Scheme depicting the different fates of NIR-IO nanocrystals during citrate concentration-dependent synthesis.

Recently, Chen et al. reported that IO nanocrystals of higher crystallinity provided better NIR absorption, better temperature increase, and higher PTA efficacy because these highly crystalline IO nanocrystals had a preferred plane orientation that resulted in effective absorption of NIR irradiation 49. In our experiments, we also observed that the NIR optical absorbance of NIR-IO nanocrystals increased when their crystallinity increased. Although the NIR optical properties of NIR-IO nanocrystals might be dominated by their crystallinity, the FT-IR spectra still indicated that citrate was bound to the surfaces of the NIR-IO nanocrystals. The UV-Vis-NIR spectra also showed an obvious alteration in the absorption of NIR-IO nanocrystals at wavelengths of 600-900 nm. Herein, based on these findings, we interpreted ligand-assisted synthesis method to grow NIR-IO nanocrystals (Figure 3). The ferrous ions in the excess citrate environment were protected by ligand chelation, preventing these ions from forming nuclei (seeds). In the crystal growth process, these nuclei would be hydrolyzed due to citrate adsorption, thereby inhibiting the growth of nuclei 25, 42. This ligand chelation effect could result in the production of smaller, amorphous particles. When lower amounts of citrate were used, the primary particles could escape citrate protection and enter the crystal growth phase, forming bulk-phase crystalline nanocrystals 43. Interestingly, two types of NIR-IO nanocrystals also revealed different NIR optical properties. This phenomenon might be caused by an alteration of the crystalline and surface ligand effects. There have been several methods reported to prepare IO nanoparticles with photothermal ability via water phase synthesis using the Fe^2+^/Fe^3+^ co-precipitation along with limited discussion causing the photothermal ability. On other hand, the IO nanoparticles prepared via partial oxidation have limitations for the utilization in biomedical applications, including difficult to control the size distribution and maintain the stability in the blood or microenvironment 51. Hui et al. synthesized stable IO nanoparticles in aqueous via partial oxidation method; however, the optical property in the NIR-region was not investigated 52. Gao et al. prepared IO nanoparticles with different size (60-310 nm) via solvothermal reaction and the results showed that the IO with larger particle size had higher photothermal ability from the accumulation of different amount of nanoparticles in the tumor area. In addition, optical properties and heating curves were similar for these different sizes of nanoparticles 53. In this study, we created a ligand-assisted co-precipitation, partial oxidation method to synthesize IO nanocrystals with NIR absorption with high stability in the aqueous enviroment. Ligand (citrate) amounts would also influence on the crystalline of IO nanocrystals and optical properties in NIR region although detail mechanism needs to be investigated.

### 2.2 Evaluation of the photothermal ability of NIR-IO nanocrystals

**Figure 4:**
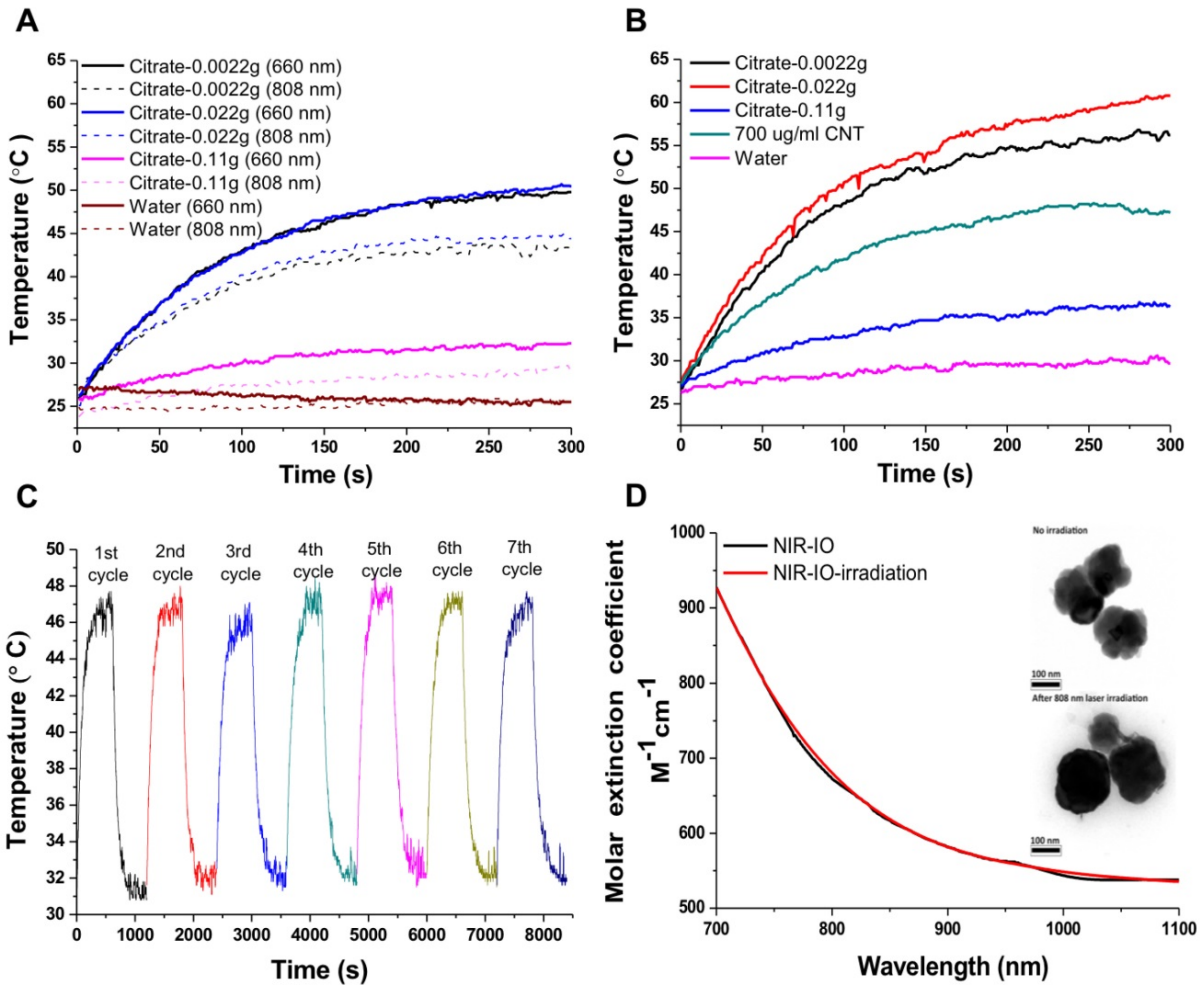
Photothermal effect of different types of NIR-IO nanocrystals after irradiation by 808- and 660-nm lasers. A) The laser power density (808/660 nm) was 0.2 W cm^-2^; B) the laser power density (808 nm) was 1.5 W cm^-2^, and the concentrations of NIR-IO nanocrystals at the same Fe concentration (350 μg mL^-1^). C) The heating curves of NIR-IO NCs with seven laser on/off cycles (1 cycle is 1 W cm^-2^, 600 s, and the concentrations of NIR-IO nanocrystals is 150 μg mL^-1^); D) Molar extinction coefficient - wavelength diagram and TEM image (insert) of NIR-IO nanocrystals with 808 nm laser irradiation or no irradiation.

NIR-IO nanocrystal revealed 182.7 nm in hydrodynamic size with PDI 0.252 in water solution and less than 5% variation in hydrodynamic diameter of NIR-IO nanocrystals was measured after 15 days. Similar results were oberved in PBS solution (Figure S3). To evaluate the photothermal effect, suspensions of NIR-IO nanocrystals prepared with different amounts of citrate were irradiated with 808 and 660 nm lasers (0.2 W/cm^2^). Figure 4A shows that irradiation at 660 nm (dashed lines) produced a slightly larger temperature increase than irradiation at 808 nm (solid lines). This difference was due to the larger transition probability of ^6^A_1_ ↔ ^4^T_2_ of Fe^+3^ ions, shown by the fact that the *ε* values were higher at 660 nm than at 808 nm. However, for cancer therapy or other biomedical applications, longer irradiation wavelengths are preferred due to their better tissue penetration. Therefore, we further evaluated the photothermal ability of the particles using higher doses (1.5 W/cm^2^) of 808-nm laser irradiation. Water and CNT were used as controls and positive controls, respectively. As shown in Figure 4B, NIR-IO prepared with 0.022 g of citrate showed the highest temperature increase (from room temperature to 60 °C) with 808-nm irradiation, whereas no obvious temperature increase occurred in the water experimental group. Notably, a two-fold higher concentration of CNT at the same light dose only increased the temperature to 47 °C. This trend was also shown in the ε values (Table 1); the ε values of all the tested NIR-IO nanocrystals were higher than that of CNT (13.51 M^-1^ cm^-1^). When the citrate amount during synthesis was increased to 0.11 g, the highest temperature attained using the NIR-IO nanocrystals decreased from 60 °C to 35 °C. As shown in Table 1, the ε value of 75.63 M^-1^ cm^-1^ for NIR-IO nanocrystals prepared with 0.11 g citrate was lower than the value of approximately 650 M^-1^ cm^-1^ found for crystals prepared with 0.022 g citrate. The photothermal conversion efficiency of the crystals was also investigated, as in previous reports^54^, ^55^; the details conversion efficiency are provided in the Supporting Information. The resulting estimate of the photothermal conversion efficiency of NIR-IO nanocrystals was 21.2%. This value exceeds that of many materials currently used in PTT, such as indocyanine green (15.1%)^56^, commercial gold nanoshells (13%)^57^ and Fe_3_O_4_@Cu_2-x_S nanoparticles (16%)^58^, and it is comparable to that of Cu_2-x_Se nanocrystals (22%)^57^, commercial gold nanorods (21%)^57^, fabricated Fe_3_O_4_ cluster-structured nanocrystals (20.8%)^59^, and polypyrrole-coated gold metal balls (24%)^60^. The NIR-IO nanocrystals also displayed rapid heating capability due to their high photothermal conversion efficiency (41%), as indicated by the time required to reach 90% of their highest temperature. These results not only demonstrated that NIR-IO nanocrystals have more potential as a promising PTT agent than indocyanine systems but also showed that NIR-IO nanocrystals have an ability similar to that of gold nanorods to serve as PTT agents due to their similar photothermal conversion efficiency. In the evaluation of photostability of NIR-IO nanocrystals, the curves of temperature raising were no obvious change after seven repeated laser irradiation cycles (light dose of 1 cycle is 1 W cm^-2^, 600 s) (Figure 4C). The results of TEM and absorption spectrum also showed no obvious change in the morphology and optical property of NIR-IO nanocrystals via seven cycles of laser on/off (Figure 4D). Photostable issue in several photothermal agents such as gold nanorods was previous reported. Zhou et al. showed the gold nanorods obvious decrease in the curves of temperature raising and optical property via laser irradiation with lower light dose (1 cycle is 0.38 W cm^-2^, 600 s). The morphology of gold nanorods also changed after laser irradiation 26. Thus, our prepared NIR-IO nanocrystals have excellent photostability for biomedical applications.

### 2.3 MRI evaluation of NIR-activated IO Nanocrystals

The NIR-IO nanocrystals produced using different amounts of citrate were studied by vibrating sample magnetometry (VSM) at room temperature. The magnetic hysteresis loops (Figure S4A) revealed that the saturation magnetization of citrate-0.11 g, citrate-0.022 g, and citrate-0.0022 g was 7.2266, 61.350, and 34.29 emu/g, respectively. This result indicates that the amount of citrate used in the synthesis of the particles also affects the magnetic properties of the particles, and the high saturation magnetization values of these three experimental groups indicates that the crystals have potential for use as an MR imaging reagent 61. To evaluate the MR ability, we chose the citrate-0.022 g experimental group. In general, magnetic nanocrystals are larger secondary structural superparamagnetic Fe_3_O_4_ particles can be used as negative MR contrast agents due to their ability to shorten the spin-spin (*T*_2_) proton relaxation time, thereby causing signal reduction and enhancing the darkness in *T*_2_-weighted images 62. Figure S4B shows the inverse relaxation times (1/*T*_2_) of the citrate-0.022 g NIR-IO nanocrystals and commercial Resovist^®^. The *T_2_*relaxation time of citrate-0.022 g NIR-IO nanocrystals was not as good as that of Resovist^®^. The transverse relaxivity (*r*_2_) constant was calculated from the slopes of the relaxation rates of 1/*T*_2_ plotted against Fe concentration. The *r*_2_ values of the citrate-0.022 g NIR-IO nanocrystals and Resovist^®^ were 141.40 and 240.51 S^-1^ mM^-1^, respectively. Although these results indicate that Resovist^®^ has better *T_2_*MR imaging ability, the MR images with 0.022 g citrate NIR-IO nanocrystals were obtained on a 3 T clinical MR scanner. The results show that the NIR-IO nanocrystals produced a concentration-dependent decrease in the signal in the MR images (Figure S4C); additionally, the nanocrystals, like Resovist^®^, could darken *T_2_*-weighted MR images in a similar manner (Figure S4C). Thus, the NIR-IO nanocrystals of 0.022 g citrate group may have potential as a promising MR enhancement agent (*T_2_*-lowering ability).

### 2.4 *In vitro* photothermal effect and cell viability study

To evaluate the photothermal effect and the toxicity of NIR-IO nanocrystals *in vitro*, HT-29 (human colorectal adenocarcinoma) cells were incubated with various concentrations of NIR-IO nanocrystals (prepared with 0.022 g of citrate) for 24 h. The cellular uptake of the nanocrystals is shown in Figure S5. The dark field microscopy images indicate that after 24 h of incubation, large amounts of NIR-IO nanocrystals were taken up by HT-29 cancer cells and distributed in the cytoplasm (red arrows).

**Figure 5:**
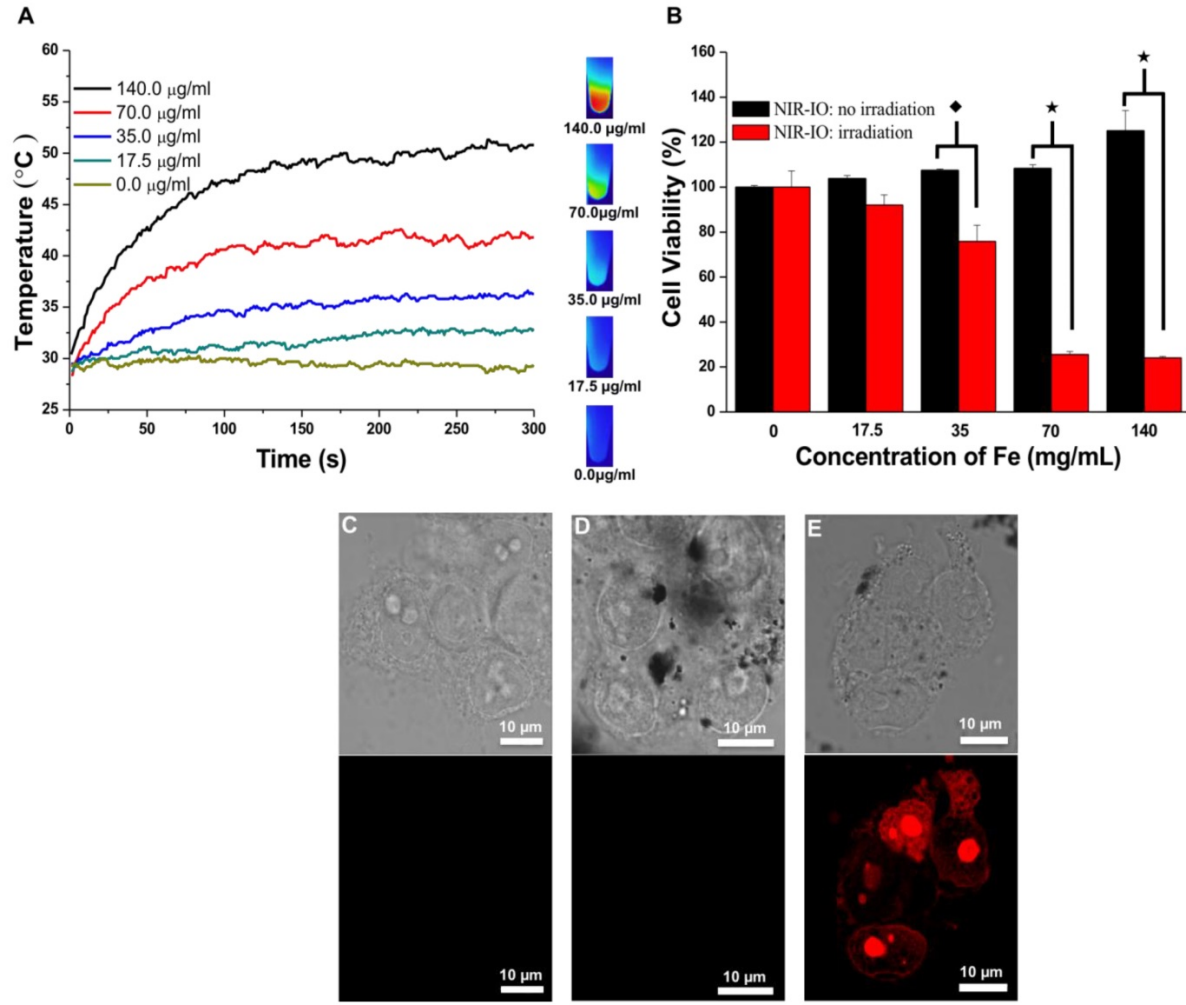
A) Heating curves of HT-29 cancer cells treated with NIR-IO nanocrystals at various Fe concentrations as a function of irradiation time using 808-nm laser irradiation (the power density was 1.5 W cm^-2^). B) Cell viability of HT-29 cancer cells incubated with NIR-IO nanocrystals at various Fe concentrations. (★) Significant difference between the group of NIR-IO irradiation and the group of NIR-IO no irradiation (*p*<0.001). (◆) Significant difference between the group of NIR-IO irradiation and the group of NIR-IO no irradiation (*p*<0.05); n = 3. Confocal laser scanning microscopy images of HT-29 cancer cells treated with C) PBS + 808-nm laser irradiation, D) NIR-IO nanocrystals and E) NIR-IO nanocrystals + 808-nm laser irradiation at a power density of 1.5 W cm^-2^. The cells were stained with PI. The Fe concentration of the NIR-IO nanocrystals was 140 μg mL^-1^.

At specified times of incubation; the cells were exposed to an 808-nm laser at a power density of 1.5 W/ cm^2^ for 5 min. The cell survival efficiency was then determined using the alamarBlue^®^ assay. As shown in Figure 5A, under 808-nm laser irradiation, the temperature increments were concentration dependent; after 5 min of irradiation, the temperature reached approximately 51 °C. Quantitative evaluation of the results (Figure 5B) showed over 90% cell survivals when the cells were exposed to either NIR-IO nanocrystals or 808-nm laser irradiation alone. Significant phototoxicity of NIR-IO-treated cells was observed; after cells were exposed to 70 µg mL^-1^ particles and 808-nm laser light irradiation for 5 min at a power density of 1.5 W/cm^2^, the cell viability decreased to 25%. These results show that the NIR-IO nanocrystals also possess excellent ability to convert NIR light to heat and to cause cell death after uptake into the cell. The increased cell viability in the group exposed to NIR-IO nanocrystals might have been caused by the availability of additional free iron released from the NIR-IO nanocrystals in the cells, which may have increased the cellular metabolic activity 63.

Photothermally treated cells were stained with propidium iodide (PI) for further investigation of the photothermal effects of the NIR-IO nanocrystals in HT-29 cells. The confocal microscopy images in Figure 5C showed that most of the cells treated with PBS plus laser irradiation retained a healthy appearance, and no red fluorescence of the PI dye was detected in the cells treated with NIR-IO nanocrystals and without laser irradiation (Figure 5D). Cells treated with NIR-IO nanocrystals with 808-nm laser (1.5 W cm^-2^) light exposure for 5 min revealed PI fluorescence accumulation in the nuclei, indicating cell membrane disruption and necrotic reactions with PTT effects (Figure 5E). The major cause of necrosis of the cells treated in this manner has been shown to be rapid heating and increased duration of heat shock.^64^ For example, higher laser power intensity irradiation produces rapid heating, causing the cell to lose its membrane integrity. Thus, our results also provide evidence concerning the photothermal effects of the NIR-IO nanocrystals taken up by cancer cells; upon laser exposure, cell structures were seriously damaged due to the rapid temperature increase caused by the excellent heat generation of the particles, causing the cells to undergo necrosis instead of apoptosis 38.

### 2.5 *In vitro* biochemical investigation after photothermal therapy

Although our investigation of the cell responses to the photothermal effect was complete, the biomolecular alterations in the treated cells were still unclear. Therefore, we used synchrotron-based infrared microspectroscopy (SR-IMS), an ultra-high spatial resolution technique, to evaluate possible alterations in biomolecules 65.

To investigate biochemical alterations in the NIR-IO-treated cancer cells, we first scanned the IR spectrum of HT-29 cancer cells in the mid-infrared range (3600-950 cm^-1^). The entire range of the spectrum is shown in Figure S6A. Figure S6B-D compares the FT-IR spectrum of HT-29 cancer cells with that of HT-29 cancer cells that given NIR-IO-mediated PTT in the three spectral regions of 3000-2800 cm^-1^ (signals from lipids), 1700-1450 cm^-1^ (signals from amide I and II), and 1300-900 cm^-1^ (signals from DNA/RNA and glycogen). There are differences in the spectral regions corresponding to lipids and to amide I and II. The mapping images shown in Figure S7 not only provide information on the distribution of these biomolecules in the cell but also show the differences in cell morphology caused by NIR-IO PTT. These results could reflect the alteration of biomolecules in HT-29 cancer cells. In summary, our preliminary results showed that NIR-IO-treated cells underwent changes corresponding to the signal of the lipid and amide I and II regions compared with untreated cells; however, there were no obvious variations in the DNA/RNA or glycogen regions of the treated cells.

The mechanisms of cell death undergo necrosis and apoptosis after PTTtreatments has been reported 66. The apoptosis pathway involves lysosomal membrane permeabilization followed by the release of cathepsins into the cytosol. The cathepsins cleave Bid and trigger the intrinsic pathway of apoptosis. Although apoptotic cells can be transformed into necrotic cells via secondary necrosis^67^, the investigation of the molecular mechanism of cell death remains an important issue in the clinical development of PTT. Pino et al. indicated that the PTT should consider the parameters (nanoparticle type, dosage, and heating capacity) needed to produce maximal apoptosis of cancer cells and thereby achieve high efficacy treatment. In our case, we also observed that NIR-IO nanocrystals were able to permeabilize the lysosomal membrane after low-intensity (0.8 W cm^-2^) 808-nm laser irradiation (data not shown). However, we chose to use high-intensity laser irradiation because of the decrease in the drug uptake into cells in an *in vivo* environment and the attenuation of the light intensity in the treated tissue.

**Figure 6:**
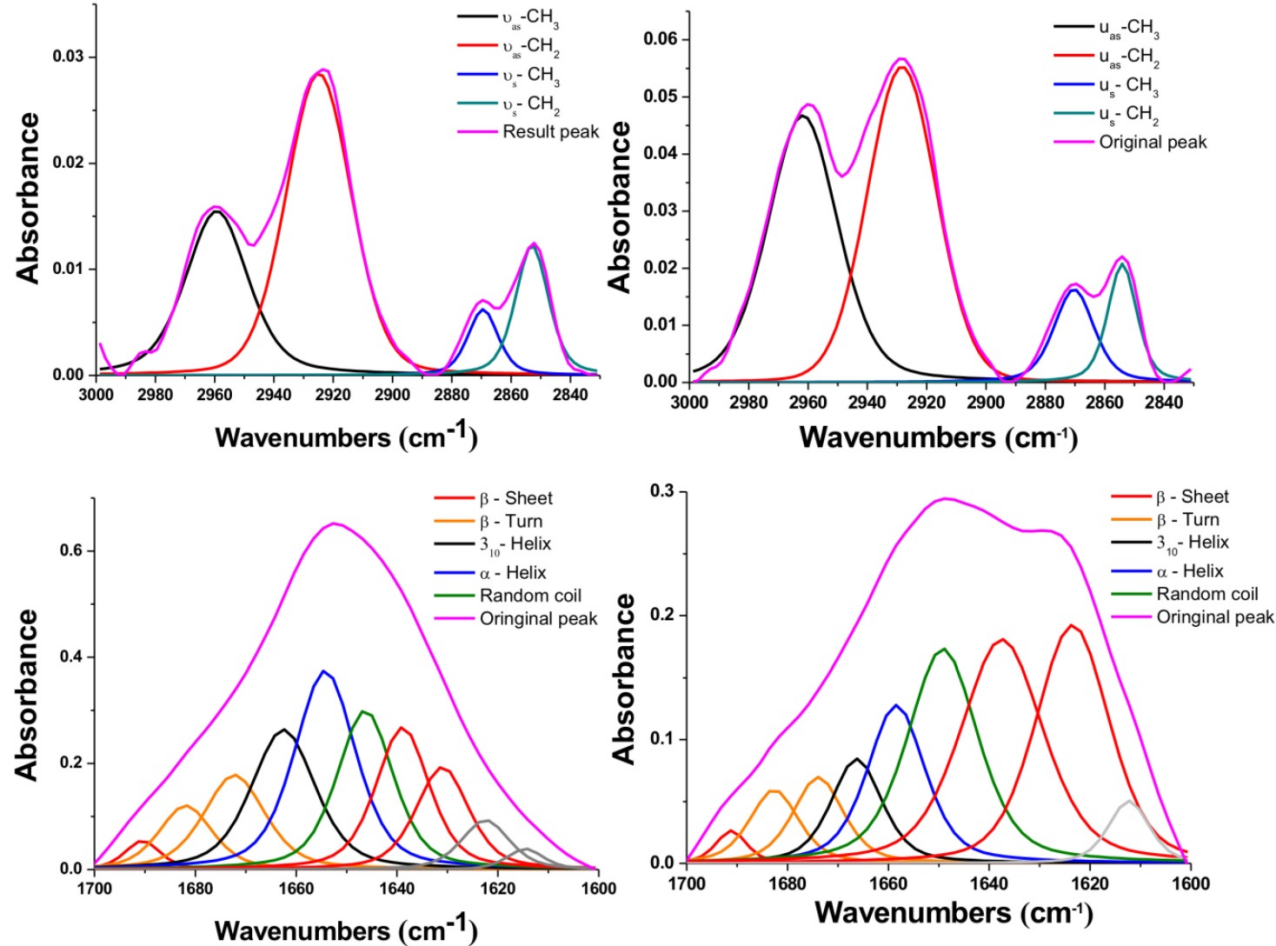
FTIR spectra of HT-29 cancer cells treated with NIR-IO nanocrystals (left) and of cells treated with NIR-IO nanocrystals + 808-nm laser irradiation (right); deconvolution analysis and Voigt peak fitting were performed in accordance with the literature.

To further study these interesting variations after NIR-IO treatment, we performed peak deconvolution of the lipid and amide I and II signals.^68^ The upper part of Figure 6 shows the region of the signal corresponding to symmetric and asymmetric stretch of -CH_2_ or -CH_3_; the signal ratios of symmetric and asymmetric -CH_3_/-CH_2_ also show obvious enhancement after NIR-IO-mediated PTT. Although the -CH_3_ and -CH_2_ signals usually indicate lipid content 69, 70, the -CH_3_ signal also has another meaning in this experiment. The -CH_3_ lipid signal had a smaller contribution than the -CH_2_ lipid signal. The -CH_3_ signal also provides information about the protein content of cells, which may change when cells experience stress. Therefore, the observed -CH_3_ signal increase after PTT may be indirect evidence that the intracellular protein content increased due to the production of heat shock proteins. As many studies have indicated, heat shock proteins (HSPs) such as HSP70 and HSP90 are generated when cells are exposed to high temperature (≥43 °C).^69^, ^71^-^73^ The investigation of the amide I and II signal region (Figure 6, lower part) showed that the intracellular protein content was altered. This phenomenon might be caused by the generation of numerous HSPs, resulting in alterations in intracellular protein secondary structure 74. Another possibility is that the high temperature condition caused α-helix unfolding while allowing β-sheets to remain intact 75. However, to produce a complete loss of secondary structure, the cells would need to reach a very high temperature (above 60 °C); this is likely why some of the α-helix signal remained after PTT 76. These results provide evidence regarding the intracellular biochemical condition and indicate that proteins underwent alterations after cells were exposed to PTT.

### 2.6 *In vivo* performance of NIR-IO nanocrystals via intratumor injection

Before moving forward to investigate magnetic, *in vivo*, tumor-targeting photothermal therapy using NIR-IO nanocrystals, we first tested the photothermal properties of the nanocrystals by intratumor injection. The spatial temperature distribution in the tumor area during NIR-IO nanocrystal-mediated PTT was measured using a thermographic camera. The body temperature of the mice prior to PTT was approximately 32 °C, and the temperature of the tumor area after exposure to an 808-nm laser at a power density of 1.5 W cm^-2^ for 10 min with or without intratumoral injection of phosphate buffered saline (PBS) approached 38 °C (data not shown). The temperature alteration after intratumoral injection of the clinical Resovist^®^ agent was also investigated (Figure S8, A-B); the results showed only a slight elevation in temperature (32 to 40 °C). However, the tumor that was intratumorally injected with NIR-IO nanocrystals (7 mg of Fe per animal) and then exposed to an 808-nm laser exhibited an excellent temperature increase from 32 to 56 °C (Figure S8, A-B). Histological analysis of the tumor tissue provided further evidence of the tumor cell responses to the high temperature. As shown in Figure S8C, examination of H&E-stained tissue sections demonstrated differences in tissue morphology in the tumors of the NIR-IO and Resovist^®^ groups 4 days after laser irradiation. In the NIR-IO group, the cells in the tumor tissue were shrunken and displayed no nuclei, whereas in the group treated with Resovist^®^, the tissues exhibited normally organized cellular structure.

In addition, a 7 T MRI instrument was used for an *in vivo* evaluation of the MR imaging of the tumor site in a mouse that was intratumorally injected with NIR-IO nanocrystals. As shown in Figure S9, the post-injection site (1.530 target pixel / background pixel, gray arrow) presented obviously darkened *T_2_*-weighted MR images compared with the same tumor prior to injection (8.435 target pixel / background pixel). These results indicate that our material provided an efficient basis for PTT and MR imaging even *in vivo*. However, in clinical cancer therapy, improving drug accumulation and achieving more efficient therapy via intravenous injection are always important. Therefore, it will be necessary to verify the theranostic efficacy and to investigate the photothermal damage that occurs in tissues after intravenous injection of NIR-IO nanocrystals.

### 2.7 *In vivo* magnetically targeted photothermal therapy and its efficacy evluation

**Figure 7:**
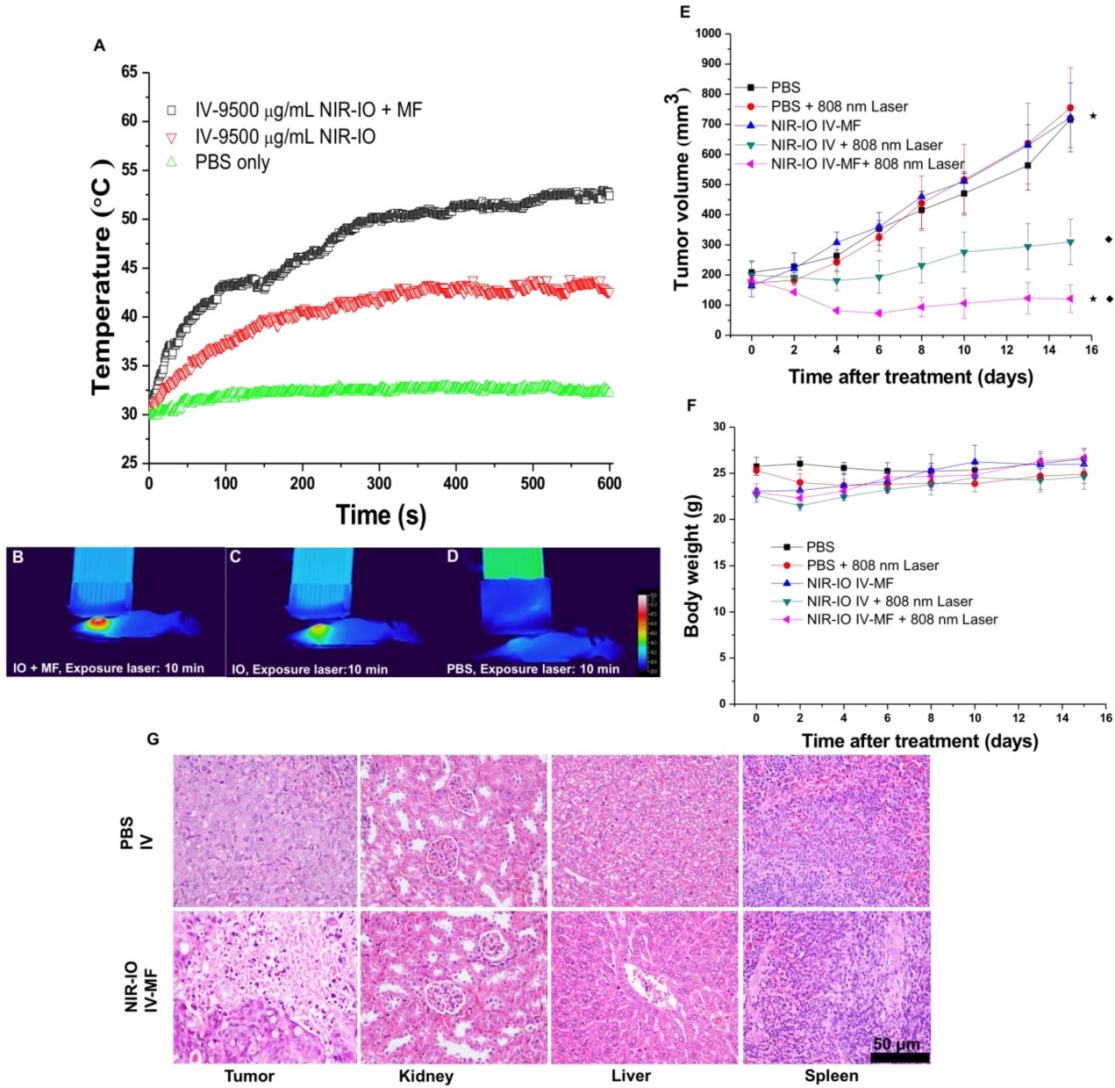
A) Heating curves; B-D) thermographic images of mice bearing HT-29 tumors that had been IV injected with PBS and NIR-IO nanocrystals (38 mg kg^-1^) after 10-min exposure to 808-nm laser irradiation (1.5 W cm^-2^) and MF targeting. E) Tumor growth, F) relative weight curves and G) H&E-stained images of HT-29 tumors in nude mice after PTT treatment. The data are presented as the mean ± standard deviation (SD). (★) Significant difference between the group of NIR-IO IV-MF + 808nm laser and the PBS group (*p*<0.001). (◆) Significant difference between the group of NIR-IO IV-MF + 808nm laser and the group of NIR-IO IV + 808nm laser (*p*<0.05); n = 3. The Fe concentration of the intravenously injected NIR-IO nanocrystals was 38 mg kg^-1^, and the 808-nm laser (1.5 W cm^-2^) irradiation time was 10 min.

Because our preliminary *in vivo* data revealed the high NIR-mediated photothermal efficiency of NIR-IO nanocrystals and their good ability to enhance MR imaging, we performed magnetically targeted PTT treatment via intravenous injection using the HT-29 tumor model in nude mice. The tumor-bearing mice were divided into five groups: PBS; PBS + 808-nm laser irradiation; NIR-IO + MF targeting; NIR-IO + 808-nm laser irradiation; and NIR-IO + MF targeting + 808-nm laser irradiation followed by intravenous injection of 100 µL of NIR-IO nanocrystals (9.5 mg mL^-1^ of Fe) and PBS. A magnet was used to magnetically target each tumor for 24 h. After magnetic targeting, the tumors with or without exposure to an 808-nm laser at a power density of 1.5 W cm^-2^ for 10 min were measured. As shown in Figure 7 (A-D), the spatial temperature distribution at the tumor surface in the MF and non-MF groups was increased to 53 °C and 43 °C, respectively. In contrast, the temperature at the tumor surface of the control group (intravenously injected PBS) remained below 32 °C during irradiation. Within 15 days, the tumor volumes in the NIR-IO + MF targeting + 808-nm laser irradiation group decreased from 183.1 ± 18.2 mm^3^ to 121.6 ± 45.4 mm^3^ (tumor growth inhibition, TGI% = 83.1) (Figure 7E). The tumor volumes in these animals were significantly smaller than those of the NIR-IO + 808 nm (TGI% = 56.8, *p*<0.05) and NIR-IO + MF groups (*p*<0.001). No significant differences in tumor volume were found among the NIR-IO + 808 nm, PBS and PBS + 808-nm laser irradiation groups. Figure 7F shows the relative weight curves of the experimental mice. The weight differences among the five groups were similar, and no apparent weight loss was observed during the experimental period. In recent clinical cancer treatments, the method of achieving drug accumulation in the tumor has been particularly important for determining the treatment conditions (i.e., better accumulation could decrease the effective treatment dosage and also reduce side effects). Herein, as our results show, NIR-IO nanocrystals represent a potentially promising treatment because they not only displayed good magnetic tumor targeting ability but also produced an excellent photothermal ablation effect. Figure S10 shows photos of mice after NIR-IO treatments. One day after irradiation, the irradiation site on the side of the tumor displayed skin burns (Figure S10, middle panel). Four days later, a large eschar was found in the MF experimental group on the tumor side; small or no areas of eschar were found on the sides of the tumors in the non-MF and PBS-treated experimental group, respectively (Figure S10, right panel). Eight days after irradiation, the tumors of mice receiving NIR-IO treatment with MF and irradiation showed great tumor growth inhibition, whereas the tumors without irradiation or those in the NIR-IO treatment experimental group continued to grow uninhibited.

**Figure 8:**
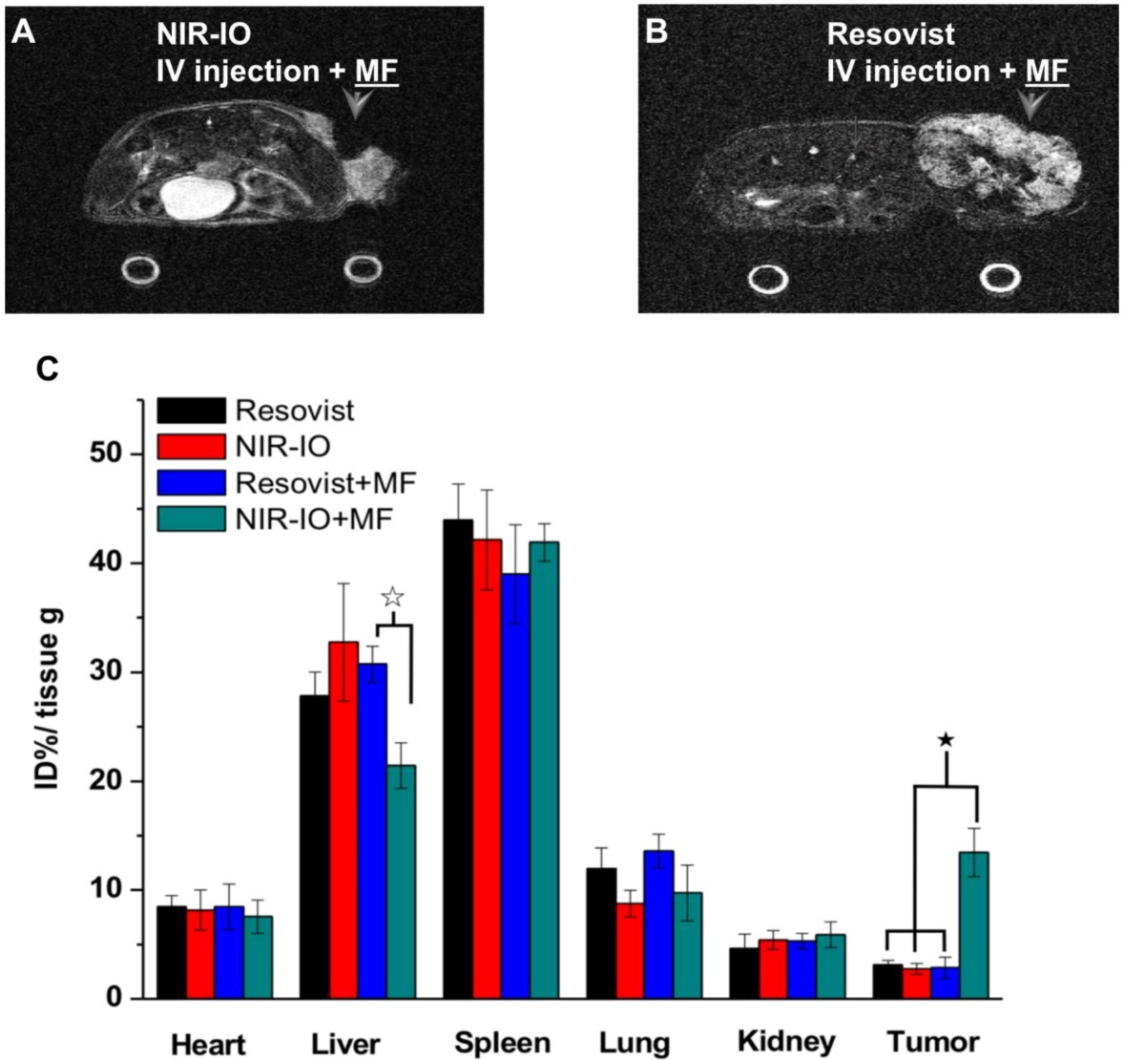
MR imaging of mice treated with A) NIR-IO nanocrystals or B) Resovist^®^ and subjected to MF targeting. In both cases, the Fe concentration was 18 mg kg^-1^. C) Biodistribution of iron amount in tumors and other organs. The data are presented as the mean ± standard deviation (SD). (★) Significant difference in tumors between the group of NIR-IO+MF and orther groups such as Resovist, NIR-IO, Resovist +MF (*p*<0.001). (☆) Significant difference in livers between the group of NIR-IO+MF and Resovist +MF (*p*<0.001). The biodistribution results was detected via ICP-OES after 24 h intravenously injection.

To further demonstrate the occurrence of photothermal damage in tumor tissue, histological methods were used 15 days after irradiation. Hematoxylin and eosin (H&E) staining of tumor tissues from the NIR-IO + MF group showed many necrotic cells that were stained by eosin, and pyknosis was also observed in the tumor tissue. The PBS group showed tumor cells with normally organized cellular structures and pleomorphic nuclei (Figure 7G). These results indicate that NIR-IO nanocrystals possess excellent ability to cause photothermal damage and cause tumor cells to undergo necrosis or apoptosis. Although photothermal damage to the tumor was clearly observed, no noticeable sign of organ damage or inflammatory lesion was observed in either the treated or untreated groups (Figure 7G). Thus, NIR-IO nanocrystal revealed high biocompatibility without irradiation and excellent PTT efficacy in irradiated area. For the evaluation of MR imaging ability via intravenous injection, NIR-IO treatment group was obviously darken than other groups in tumor region (Figure S11 and Figure 8). *T_2_*-weighted MR image of NIR-IO nanocrystals of plus magnetic targeting treatment (1.220 target pixel / background pixel) was 4.8-fold darked area in tumor area compared with that in commercial Resovist^®^ plus magnetic targeting treatment group (5.902 target pixel). Biodistribution of NIR-IO nanocrystals were also evaluated after 24 h intravenous postinjection. As shown in Figure 8C, similar tissue accumulation phenonmena were observed in Resovist^®^ and NIR-IO without MF targeting groups.NIR-IO nanocrtystals revealed significatly increased tumor accumulation and decreased liver accumulation with MF application. It is noticed that more than 4-fold higher accumulation in NIR-IO with MF targeting group were observed than in other three groups (NIR-IO without MF targeting or Resovist^®^ withour or with MF targeting groups). MF applications for increased tumor accumulation were utilized in magnetic nanocarriers. Zhang et al. designed the doxorubicin (DOX)-loaded hollow mesoporous CuS nanoparticles (HMCuS NPs) with superparamagnetic IO nanoparticles for chemo-phototherapy and 1.5-fold increment were observed in biodistribution results after MF targeting 77 Qian et al. reported the theranostic nanocapsules made by PCLA-PEG-PCLA polymers hierarchically assembling SPIOs, IR820 and a chemotherapeutic agent for tumor theranostics and 2-fold accumulation increment were observed in tumor area after MF targeting 78 Dong et al. was prepared magnetic mesoporous silica nanoparticles for cancer gene therapy. The biodistribution results showed that 1.5-fold enhancement in the tumor were observed via MF targeting strategy 79 Guo et al. developed MF targeting strategy via IO nanoparticles and 1.94-fold increments of tumor accumulation were observed with MF targeting 80. Our prepared NIR-IO nanocrystals revealed not only excellent PTT efficacy bit also more than 4-fold enhancement in tumor accumulation with MF targeting strategy. The reduced liver accumulation in NIR-IO with MF tageting group also support our prepared NIR-IO nanocrystals for cancer theranostics (Figure 8C). Krishnan et al. reported detail situations such as blood clearance pharmacokinetics, biodistribution studies with size effect, and metabolic pathway of IO nanoparticles in living body.^81^ We proposed that our prepared NIR-IO nanocrystals may form the protein-nanocrystal complex in the circulation and results in higher accumulation in spleen/liver and lower accumulation in kidney (Figure 8C). Aforementioned situations would cause by mechanical filtration of macrophage phagocytosis in spleen 81, 82. The fate of liver accumulations of NIR-IO nanocrystals would result in the cellular uptake by Kuppffer cells and then iron-based nanocrystals would be lysed in acidic organelles such as lysosomes. The similar mechanism would happen in spleen accumulation; however, the degradation rate via macrophages was slower in spleen than Kupffer cells in liver tissue due to the less iron storage proteins presence in the spleen. In addition, the injection dose would also influence on the degradation rate. Wei et al. reported that the metabolism of high dose IONPs (8.4 mg kg^-1^) needed near three weeks for degradation.^83^ Thus, we speculated that our prepared NIR-IO nanocrystals may need more than one month for degradation. Recently, some researchers reported that the IONPs would cause inflammatory factors such as IL series or TNF-α, however no obvious tissue toxicity was observed in the spleen 84. We also investigated the relationship of tumor accumulation of NIR-IO nanocrystals and its photothermal efficacy *in vitro* and *in vivo*. The photothermal profile of NIR-IO + MF targeting with laser irradiation* in vivo* (Fig. 7A) is similar as that of 140 μg /ml NIR-IO nanocrystal *in vitro* (Fig. 5A) that may due to the PTT efficacy from similar concentration of NIR-IO.

The use of optical nanocrystals that absorb light at NIR wavelengths followed by conversion of the absorbed energy to heat have attracted much interest as a means of modulating phototreatment in deep tissues with relatively low absorption/scattering at λ = 650-900 nm. This photothermal conversion process potentially minimizes thermal injury to the surrounding tissues.^66^, ^67^ NIR IO nanocrystals display a promising combination of optical and magnetic features in a single Fe-O compound, making their optical application easy to integrate with magnetically targeted accumulation in the area of interest. Some very recent studies have examined photothermal cancer therapy using IO nanocrystals. As shown in Table 2, Chu et al. used co-precipitation synthesis to prepare Fe_3_O_4_ nanoparticles that were used in PTA in tumor-bearing mice^39^, Espinosa et al. used the thermal decomposition method to prepare Fe_3_O_4_ nanoparticles with a low light dose (180 J cm^-2^) as a PTT agent^85^, and Shen et al. designed a cluster of Fe_3_O_4_ nanoparticles that were used at a low injection dosage against A549 tumors 86. However, the above-mentioned studies evaluated the PTT efficacy using the intratumoral injection model. Therefore, these evaluations are limited in their clinical application. In addition, Zhou et al. designed Fe/Fe_3_O_4_ nanoparticles (1460 mg [Fe] kg^-1^ body weight) to be used against HeLa tumors. This research used an MF targeting strategy to achieve higher Fe/Fe_3_O_4_ nanoparticle accumulation in tumors, achieving enhanced MR contrast images and excellent therapy efficacy after 808-nm laser (93 J cm^-2^) irradiation for 14 days 26 Chen et al. used the thermal decomposition method to synthesize Fe_3_O_4_ nanocrystals with high lattice orientation along the (400) and (440) planes. The animal experimental results showed that these nanocrystals yielded promising PTT efficacy at a dosage of 20 mg [Fe] kg^-1^ body weight and a light dose of 1500 J cm^-2^.^49^ Ren et al. reported the red blood cell-derived membrane vesicles for Fe_3_O_4_ nanoparticles encapsulation and their *in vivo* results showed that PTT efficacy at a dosage of 2.5 mg [Fe] kg^-1^ body weight with a light dose of 1500 J cm^-2^ 87 Yang et al. also evaluated PTT effects of hyaluronan-modified IONPs at adosage 20 mg [Fe] kg^-1^ body weight with a light dose of 1200 J cm^-2^ 88. Although the above-mentioned studies displayed several advantages in PTT efficacy, some limitations (i.e., in treatment too high doses or light dose) were also raised in these studies. Notably, Shen et al. provided a hydrothermal method to synthesize Fe_3_O_4_ nanoparticles with high biocompatibility and stability and these Fe_3_O_4_ nanoparticles showed not only great enhancement of MR contrast images (70-100 mg [Fe] kg^-1^ body weight) but also good PTA ability (450 J cm^-2^) via MF targeting.^38^ Although they did not directly clarify the mechanisms for the NIR absorption ability, the synthesis process indirectly provided evidence for a relationship between the ligand-assisted synthesis approach and NIR absorption features. Additionally, Guo et al. used different size of IO nanoparticles prepared by hydrothermal method for* in vivo* PTT efficacy; however the multiple procedure of PTT (drug injection at days 0, 3, 6 and laser irradiation at 1, 4, 7) was not easy to be carried out in clinical studies.^53^ Recently, ferroptosis is a new pathway of iron-related cell death 89 90. It is also reported that the median lethal dose (LD-50) of IO nanoparticles was 300-600 mg [Fe] kg^-1^ 81. Thus, we used the less dosage of NIR-IO (38 mg [Fe] kg^-1^) in this study that is superior biocompatibility and more safety for further biomedical applications.

Other inorganic nanoparticles, including carbon nanostructures^91^, quantum dots^92^, and gold nanoparticles^33^, can also be used for PTT. Our results provide evidence for an excellent magnetic targeting effect of the NIR-IO nanocrystals and diminished distribution of these nanoparticles in other reticuloendothelial system (RES) organs. Additionally, the IO nanoparticles have Federal Drug Administration approval for clinical use.^21^, ^39^, ^93^ Additionally, the clinical applications and studies of magnetic thermal therapy still foucus on hyperthermia model, which is a heat producing therapy via applying an external magnetic field acting on superparamagnetic iron oxide nanoparticles (SPIO) 94. This model had entered clinical studies via MagForce (Berlin, Germany) in 2007, and recent clinical trials began to evaluate the hyperthmia therapy in patients with recurrent glioblastoma multiforme and metastatic bone tumors.^95^ However, there were many cases or studies of clinical trails in the area of (SPIO) nanoparticles hyperthermia therapy, the limitations of this therapy still existed and must to be solved. The major clinical limitation of SPIO nanoparticles was too high treatment dosage 96, 97 ([Fe] are usually over 1 M in the phase I study, which is almost or over ten folds than the dosage of this study used) due to SPIO nanoparticles had lower specific loss power (SLP, or heat dissipation per unit mass of magnetic material).^98^ Although, SPIO nanoparticles with higher SLP value (near 1000 W/ g range) had been reported in the recent years, the heating performance of these SPIO nanoparticles had to reached its limit 85. Therefore, to concern the clinical dosage and treatment efficacy, the photothermal therapy is might a potential treatment model in cancer therapy because its higher heating efficiency and lower treatment dosage. For biomedical applications, our *in vivo* results revealed the great potential of NIR-IO nanocrystals for use in clinical cancer theranostics due to the low treatment dosage and light doses required and their excellent PTT efficacy, MR contrast ability, high accumulation in the tumor area, and low cytotoxicity to other organs.

We also investigated the influence of the ligand used to synthesize the nanocrystals. The synthesis ligand citrate was replaced with a photosensitizer (dendrimer phthalocyanine, DPc). Figure S12 shows the TEM results obtained after using the photosensitizer to replace citrate. When larger amounts of the photosensitizer were added, variations similar to those observed for changes in the citrate concentration appeared. This result emphasizes the importance of the ligand-metal ion interactions, showing that compounds with groups that perform similar functions can provide the same function in our material synthesis, and it also shows that this technique can be used to prepare nanocrystals suitable for use in photodynamic or other types of therapy.

## 3. Conclusion

We used co-precipitation, partial oxidation approach to produce IO nanocrystals that display excellent NIR absorption and MR properties. Ligand effects on IO nanocrystals and crystalline of IO nanocrystals obviously influence the photothermal ability of NIR-IO. NIR-IO mediated PTT revealed efficient cancer cell membrane disruption and intracellular protein alterations for cell killing. With MF targeting in tumor area, NIR-IO by intravenous injection resulted in excellent accumulation of nanocrystals in the tumor, lower RES distribution, obvious enhancement of MR *T_2_* contrast imaging, and great photothermal ablation ability. Most importantly, during the entire treatment, no noticeable toxicity was observed, indicating the good biocompatibility of prepared NIR-IO nanocrystals. Therefore, NIR-IO nanocrystals have a promising potential for clinical photothermal cancer treatment and MR imaging.

## 4. Materials and Methods

**Materials:** The reagents used and their sources were as follows: ferrous sulfate heptahydrate (FeSO_4_**·**7H_2_O, ≥99%) (Sigma-Aldrich); trisodium citrate (99.5-100.5%, J. T. Baker); benzene-1,3,5-tri-carboxylic acid (trimesic acid (TMA), 98%) (Alfa Aesar); sodium hydroxide (NaOH, 99.0%) (Riedel-de Haën); ethanol (EtOH, 99.9%) (J. T. Baker); hydrazine monohydrate (N_2_H_4_**·**H_2_O, 98%) (Sigma-Aldrich); sodium nitrate (NaNO_3_, ≥99.5%) (Sigma-Aldrich); and dendrimer phthalocyanine (DPcZn, a 2^nd^ generation acrylic ether dendrimer with zinc(II) in the phthalocyanine center and 32 carboxylic groups on its periphery) (a generous gift from Professor Kazunori Kataoka, University of Tokyo, Japan).

**Preparation of Citrate-Cojugated NIR-IO Nanocrystals:** Eighty milligrams of FeSO_4_**·**7H_2_O and trisodium citrate (110, 22, 2.2 mg) were dissolved in 15 mL of deionized water with stirring, immediately transferred to N_2_ flask and purged with nitrogen for 10 min. The solution was then heated to 85 °C, and 0.377 M NaOH solution containing 0.0185 mM TMA and 1.2 M N_2_H_4_ was added dropwise. The rate of addition of NaOH was 2 mL min^-1^. After the NaOH addition, the solution was allowed to react for 1 hour, during which time the color changed from brown to black. The solution was then cooled to room temperature, and the as-synthesized IO products were removed by dialysis for 4 days against deionized water and separation via magnetic attraction. The IO products were then dispersed in 5 mL of deionized water for future use.

**Preparation of DPcZn-Conjugated IO Nanocrystals:** This preparation process used DPcZn to replace citrate: 8 mg of FeSO_4_**·**7H_2_O and various amounts of DPcZn (9.9 or 66 mg) were dissolved in 15 mL of deionized water with stirring and protection from light, then immediately transferred to N_2_ flask and purged with nitrogen for 10 min. The solution was then heated to 85 °C, and 0.377 M NaOH solution containing 0.00185 mM TMA and 0.12 M N_2_H_4_ was added dropwise. The nanocrystals formed during this process were purified as described above.

**Photothermal Effect Evaluation for 808-nm and 660-nm Laser Irradiation:** The concentrations of Fe in the Fe_3_O_4_ nanoparticle solutions were determined quantitatively by absorbance spectrophotometry. After the concentrations of the solutions were determined, 100 µL of solutions containing various concentrations of Fe_3_O_4_ nanocrystals were placed in Eppendorf tubes. Then, 808- and 660 nm continuous-wave (CW) diode lasers (the power density of the 808-nm laser was 1.5 W cm^-2^ , and that of the 660-nm laser was 0.2 W cm^-2^) were used to irradiate the solutions. The temperature was measured using a thermographic camera (TVS-500 EX, NEX Avio Infrared Technologies Co., Ltd.).

**Cell Line and Cell Culture Conditions:** In the *in vitro* studies, HT-29 human colorectal adenocarcinoma cells (ATCC No: HTB-38^TM^) were used. HT-29 cells were grown in T-75 flasks (BD Falcon) in Dulbecco's modified Eagle's medium (DMEM; Gibco BRL, Gaithersburg, MD, USA) containing 1 mM sodium pyruvate (Gibco BRL) and supplemented with 10% fetal bovine serum (FBS; Gibco BRL) and 1% penicillin-streptomycin-neomycin solution (PSN; Gibco BRL) at 37 °C in a 5% CO_2_ atmosphere.

**Photothermal Effect, Cytotoxicity and Biochemical Molecular Studies *In vitro*:** To evaluate photothermal efficacy of NIR-activated iron oxide nanoparticle *in vitro*. HT-29 cells were seed in 6 cm diameter culture dish with culture medium. Each dish contained 10^5^ cells then put these culture dishes into incubator and waited 24 h to let cells adhesion. The cells were then treated with NIR-activated IO nanocrystals with various concentrations (140, 70, 35, 17.5 µg mL^-1^) and incubated for an additional 24 h at 37 °C. At the specified time point, the treatment medium was removed, the cells were rinsed three times with PBS. After step of washing cells with PBS, cells were collected into an eppendorf by using 0.25% trypsin-EDTA and then replaced trypsin-EDTA to fresh culture medium. The collected cells were exposed to 808 nm continuous wave diode laser (the power density of 808 nm laser is 1.5 W cm^-2^) for 5 minutes. The temperature alteration was recorded by thermographic camera. The exposed cells were planted into 96-well plates at the density of 8000 cells per well and then incubated for 24 h. To investigate the cell viability, alamar blue assay was performed. The original culture medium in each well was removed and replaced with 100 µL of culture medium containing 10 µL of alamarBlue^®^ reagent (Molecular Probes, Invitrogen). The 96-well plate was then incubated for 3 h at 37 °C, during which time the resazurin (non-fluorescent) was reduced to resorufin (fluorescent). The cell viability was quantified using a scanning multiwell ELISA reader (SpectraMax^®^ M2_e_, Molecular Devices, USA) at an excitation wavelength of 570 nm and a fluorescence emission wavelength of 590 nm. We also used propidium iodine (PI; Molecular Probes, Invitrogen) to verify the photothermal effect of NIR-activated IO nanocrystals on cancer cells. For this measurement, HT-29 cells were treated with 140 µg mL^-1^ nanocrystals and then irradiated (or not) for 5 min with a CW diode laser (808 nm) and incubated for an additional 24 h at 37 °C. The cells were then stained with PI and imaged by confocal laser scanning microscopy (CLSM; Leica-SP5, Leica Microsystems Heidelberg GmbH, Germany). For biochemical molecular investigation, HT-29 cells with or without NIR-IO-mediated PTT were seeded 5×10^4^ cells on low-e slides and incubated for 24 h, respectively. These adhered cell samples were washed using PBS, fixed using 4/% paraformaldehyde and then dried overnight for futher investigation. The Fourier transform infrared (FTIR) imaging of as-prepared cell samples were measured by synchrotron-based infrared microspectroscopy (SR-IMS; beamline 14A1, National Synchrotron Radiation Research Center, Taiwan). The spectra were recorded in reflectance mode from each sample section using a Thermo Nicolet 6700 spectrometer and a continuum infrared microscope with the following settings: mid-infrared range 3600-650 cm^-1^, resolution 4 cm^-1^, step size 10 μm, aperture 15*15 μm^2^ and 128 scans. Peak positioning, deconvolution, and baseline corrections were performed using OMNIC peak-resolving software.

**Animal and *In vivo* Examinations:** The *in vivo* experimental protocols were approved by the Institutional Animal Care and Use Committee of National Chung Hsing University (IACUC of NCHU). Female BALB/cAnN.Cg*-Foxn1^nu^*/CrlNarl nude mice (4-5 weeks old, 20 ± 2 g) were obtained from the National Laboratory Animal Center (Taiwan). The mice were kept in an air-conditioned facility under an artificial light-dark cycle and provided with standard food and filtered water. The mice were acclimated to this environment for at least three days prior to subcutaneous injection in the right hindquarter with 2 × 10^6^ HT-29 cells suspended in serum-free DMEM. The tumor size was calculated as 1/2(4π/3) (L/2) (W/2) H, where L is the length, W is the width, and H is the height of the tumor. Treatment was initiated when the tumor reached a volume of 150-200 mm^3^.

**Photothermal Effect and MRI Studied *In vivo*:** To study the photothermal effect and MRI *in vivo*, we used two different methods. Mice bearing HT-29 tumors approximately 200 mm^3^ in size were treated with 100 µL of PBS (Control) or NIR-IO (9.5 mg mL^-1^) via intravenous injection or with 100 µL of Resovist^®^ (3.5 mg mL^-1^) or NIR-IO (3.5 mg mL^-1^) via intratumoral injection for photothermal effect investigation. In the experimental group that received intravenous injection, we attached a small magnet to the tumor 24 h after the intravenous injection. At specific times thereafter, the tumors were exposed to an 808-nm NIR laser. In the intratumoral injection group, the tumors were exposed to the 808-nm NIR laser immediately after intratumoral injection. The power density of the 808-nm NIR laser (1.5 W cm^-2^ for 10 min) was the same for the intravenous and intratumoral injection groups. During irradiation, the temperature of the tissue was recorded by a thermographic camera. To evaluate the quality of magnetic resonance imaging (MRI), PBS (control; 100 µL), Resovist^®^ (100 µL; 9.5 mg mL^-1^), and NIR-IO (100 µL; 9.5 mg mL^-1^) were intravenously injected into HT-29 tumor-bearing mice. After intravenous injection and exposure to the magnetic targeting field for 24 h, the tumor-bearing mice were subjected to MR imaging using a 7 T MRI (Bruker, USA) under halothane gas anesthesia before and after injection. TurboRARE-T2 pulse sequences (TR/TE=5000 ms/56 ms, flip angle=180°, matrix size=256 x 128) were used for *T_2_*-weighted imaging. The slice thickness was 1 mm with a 1 mm gap, and the field of view (FOV) was 9 x 3.5 cm^2^ for coronal scanning.

**Antitumor Efficacy of NIR-IO Nanocrystals and NIR-IO Nanocrystals with MF Targeting- Mediated PTT:** To investigate the antitumor efficacy of our treatments, mice bearing HT-29 tumors approximately 150 mm^3^ in size were randomized into five treatment groups (n = 3 per group): PBS, PBS with laser irradiation, NIR-IO + MF targeting, NIR-IO with laser irradiation, and NIR-IO + MF targeting with laser irradiation. The animals were treated with 0.1 mL of PBS and 0.1 mL of NIR-IO (Fe_3_O_4_, 9.5 mg mL^-1^) via intravenous injection (day 0). At the end of the treatment, a small magnet was attached to the tumor of the MF targeting groups. Twenty-four hours after intravenous injection, the magnet was removed and the tumor was irradiated with an 808-nm NIR laser (1.5 W cm^-2^) for 10 min. The percentage of tumor growth inhibition (TGI%) was calculated from the relative tumor volume on day 15. The tumor size and body weight were measured every 2 or 3 days for the duration of the experiment.

**Histological Analysis of Tumor Tissue:** Tumors were excised and weighed after the mice were euthanized. For hematoxylin and eosin (H&E), the tumor tissue was fixed in formalin and embedded in paraffin. Then, paraffin-embedded 3-μm tumor sections were deparaffinized, rehydrated, and incubated in 3% H_2_O_2_ to inhibit endogenous peroxidase activity. The sections were also counterstained with hematoxylin and eosin (H&E) or hydrochloric acid (20%). The stained sections were monitored at low power (40X) and counted at high magnification (400X). Images of the stained sections were acquired using a light microscope (BX 50, Olympus) equipped with a digital camera (DP 20, Olympus) and used to analyze the damage to the tissue structure and the NIR-IO nanocrystal accumulation in the tissue.

**Biodistribution of NIR-IO nanocrystals:** The quantitative analysis of NIR-IO, Resovist, NIR-IO+MF, Resovist+MF (n = 4 per group) was analyzed in female BALB/c nude mice bearing HT-29 tumors. The agent was administered intravenously as a single dose ([Fe]: 18 mg kg^-1^). Mice were sacrificed by cervical dislocation 24 h after drug administration. Tumor, heart, liver, spleen, lung and kidney collected from each mouse and snap frozen in liquid nitrogen. These organs were lyophilized for 24 h in a freeze dryer and then weighted. The samples were then dissolved in the 20 % nitric acid for 48 h. All samples were spun at 16000 *g* in a centrifuge for 30 min and the supernatants were analyzed for iron content by ICP-OES (Thermal Fisher Scientific, iCAP^TM^ 7400). All results were normalized in the units of percentage of injected dose per gram (% ID per g) of tissue.

**Characterization of NIR-IO:** Electron micrographs were obtained using transmission electron microscopes (TEM, JEM 1400 and JEM 2010, JEOL Ltd., Japan). A UV-Vis spectrophotometer (HITACHI U-300, Tokyo, Japan) was used to determine the absorption characteristics of the samples. Fe ions were quantified using an atomic absorption spectrophotometer (GBC Scientific 932 AA, Melbourne, Australia). IR spectra were measured using a KBr plate in a FTIR spectrometer (Thermo Nicolet 6700, Madison, WI, USA). The magnetic properties of the NIR-IO nanocrystals were examined with a vibration sample magnetometer (VSM, LakeShore). The size of NIR-IO nanocrystals was measured using a Zetasizer Nano ZS apparatus (Malvern Instruments, Worcestershire, UK).

**Statistical Analysis:** The data are expressed as the mean ± SD. The tumor volumes for each group measured at different time points are summarized as the mean values and SDs. Group effects on tumor volume were tested using a linear mixed model with Bonferroni correction and are presented as the estimated marginal means (EM mean values) and the corresponding 95% confidence interval (CI) for tumor volume, with adjustment for time effects. Statistical significance was set at 0.05. Statistical analyses were performed using the SPSS 15.0 software package (SPSS Inc., Chicago, IL, USA).

## Supplementary Material

Supplementary figures and tables.

## Figures and Tables

**Table 1:** Molar extinction coefficient (ε) values at wavelength of 808 and 660 nm.

Sample	ε-value of 808nm [M^-1^cm^-1^ ]	ε-value of 660nm [M^-1^cm^-1^ ]
A-0.0022g	673.13	1027.75
B-0.022g	645.83	945.67
C-0.11g	75.63	142.49
D-CNT	13.51	16.20

**Table 2:** Efficacy comparison of photothermal cancer therapy studies that using iron oxide

Sample	Size / PTT conversion efficiency (nm) / (%)	Injection model	[Fe] injection (mg kg^-1^)	Irradiation wavelength (nm)	Light Dose (J cm^-2^)	ΔT Heating Temperature (^o^C)	Ref
Fe_3_O_4_/DSPE-PEG	10 / not offered	I.T	20-25	808	300 (1/24 Days)	not offered	38
Fe_3_O_4_ clusters of NPs	225 / not offered	I.T	2.5	808	900	22	72
Fe_3_O_4_ NCs	20 / not offered	I.T	35	808	180	15	71
HCIONPs	15 / not offered	I.V	20	885	1500	25	47
Fe/Fe_3_O_4_	13 /20.3	I.V	1460	808	93 (1/14 Days)	12	25
Fe_3_O_4_@CMCTS	177 / not offered	I.V	70-100	808	450	22	37
Fe_3_O_4_-310 nm	310 /not offered	I.V	40 (1/3 Dose)	808	270 (1/3 Days)	20	75
MNC@RBCs	200 / not offered	I.V	2.5	808	1500	12	73
HA-SPIONs	20 /not offered	I.V	20	808	1200	not offered	74
NIR-IO (ours)	156 / 21	I.V	38	808	900	22	
