# Supplementary material for: Co-precipitation Synthesis of Near-infrared Iron Oxide Nanocrystals on Magnetically Targeted Imaging and Photothermal Cancer Therapy via Photoablative Protein Denature
Source: Nanotheranostics. 2019 May 25;3(3):236–54. doi: 10.7150/ntno.24124 (PMC6584136; doi:10.7150/ntno.24124)
Supplement: Supplementary file 1 — Supplementary figures and tables. [file ntnov03p0236s1.pdf]

Supporting Information

Co-precipitation Synthesis of Near-infrared Iron Oxide Nanocrystals on Magnetically Targeted Imaging and Photothermal Cancer Therapy via Photoablative Protein Denature

Wei-Jhe Syu, Chih-Chia Huang, Jong-Kai Hsiao, Yao-Chang Lee, Yu-Tsang Huang, Parthiban Venkatesan, Ping-Shan Lai*

[*] Prof. Ping-Shan Lai, Dr. Wei-Jhe Syu, and Dr. Parthiban Venkatesan
Department of Chemistry
National Chung Hsing University
Taichung City 402, Taiwan
E-mail: pslai@email.nchu.edu.tw

Prof. Chih-Chia Huang
Department of Photonics
National Cheng Kung University
Tainan City 701, Taiwan

Dr. Jong-Kai Hsiao,
Department of Medical Imaging
Buddhist Tzu Chi General Hospital, Taipei Branch
New Taipei City 231, Taiwan

Dr. Yao-Chang Lee
National Synchrotron Radiation Research Center
Hsinchu Science Park,
Hsinchu 30076, Taiwan

Dr. Yu-Tsang Huang
Menglinbeier Clinic
Taichung City 407, Taiwan

Keywords: photothermal therapy, Fe_3O_4 nanocrystals, magnetic field targeting, near-infrared,

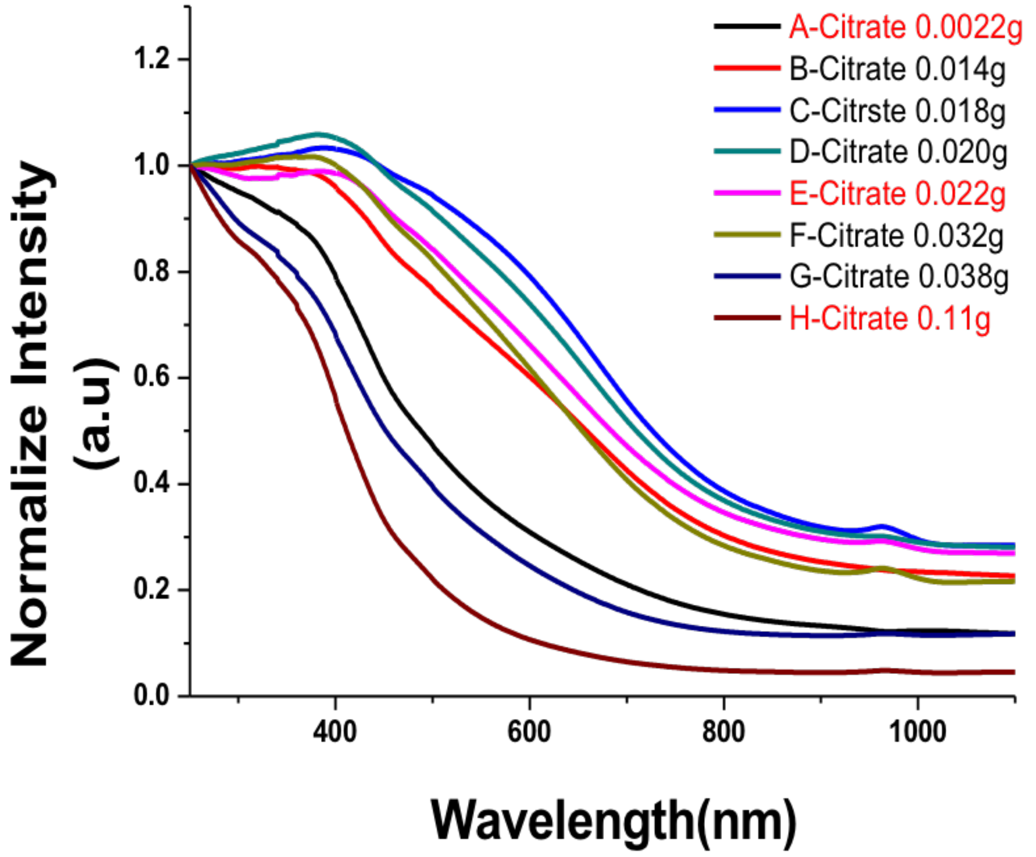

Figure S1. Absorption spectra of NIR-IO nanocrystals synthesized using different amounts of citrate.

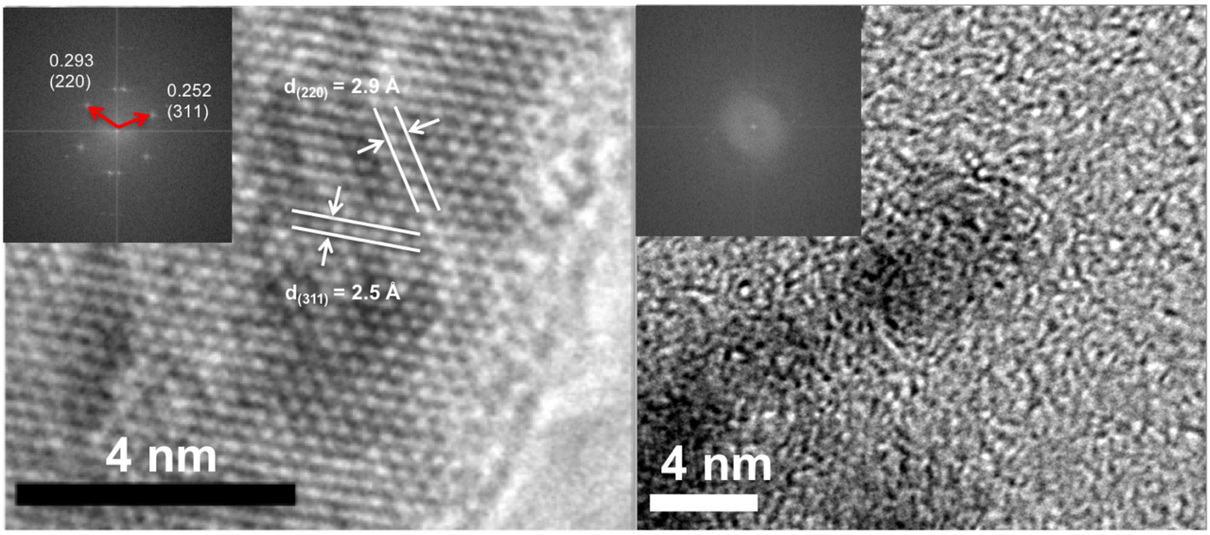

Figure S2. HR-TEM image, and fast Fourier transform (FFT) pattern of a) low citrate weight (0.022 g) synthesis, and b) high citrate weight (0.11 g).

To calculate the photothermal conversion efficiency (η) of NIR-IO nanocrystals, the following equation was used:

$$\eta = \frac{h\,A\,\Delta T_{max} - Q_{Dis}}{I\,(1 - 10^{-A_\lambda})} = \frac{m_D\,C_D}{\tau_s} \cdot \frac{A\,\Delta T_{max} - Q_{Dis}}{I\,(1 - 10^{-A_\lambda})}$$

In this equation, h is the heat transfer coefficient that can transfer to $m_D C_D(\tau_s)^{-1}$, m_D (or C_D) is the mass (or heat capacity) of water, A is the surface area of the container, ΔT_{max} is the temperature change of the sample at the maximum steady-state temperature, Q_{Dis} is the heat dissipation from the light absorbance of the solvent, I is the laser power, and A_λ is the sample absorbance at 808 nm. From this equation, the η value and 90% highest temperature η value of NIR-IO nanocrystals were calculated to be 21.2% and 41%, respectively.

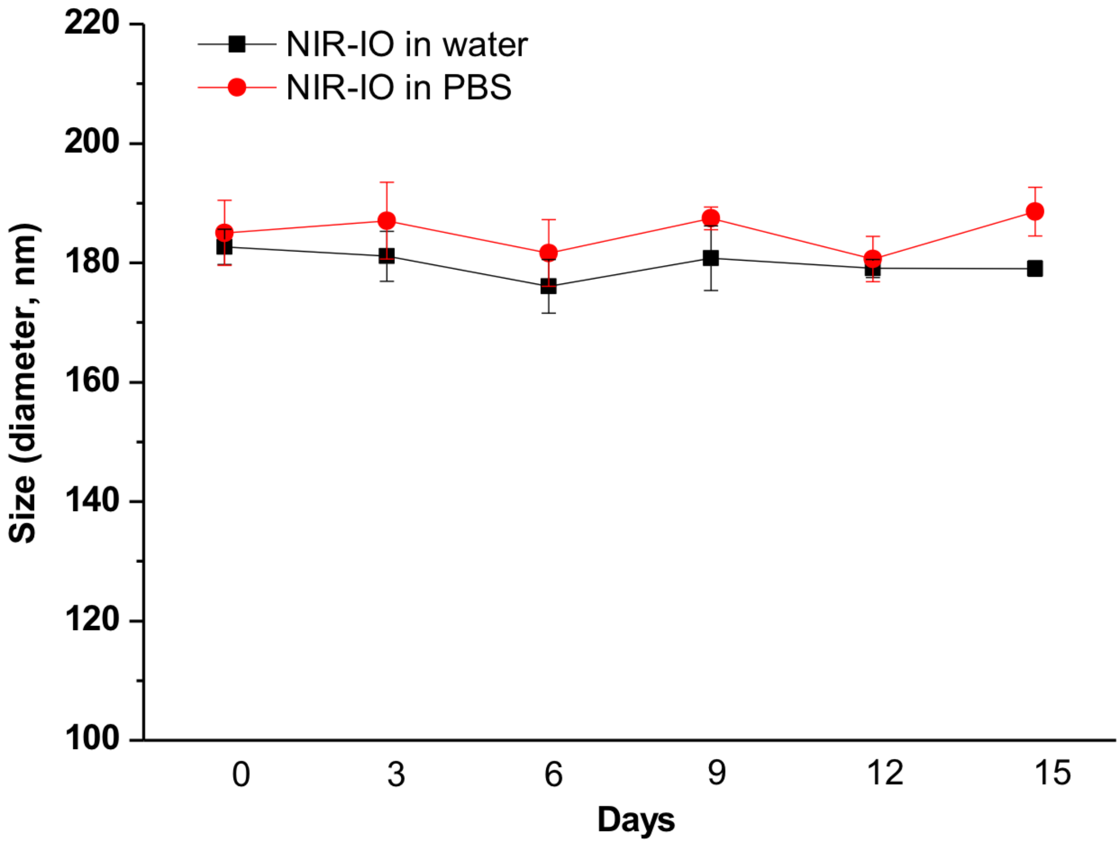

Figure S3. Size distribution of NIR-IO in the water and PBS, 1-15 days, room temperature.

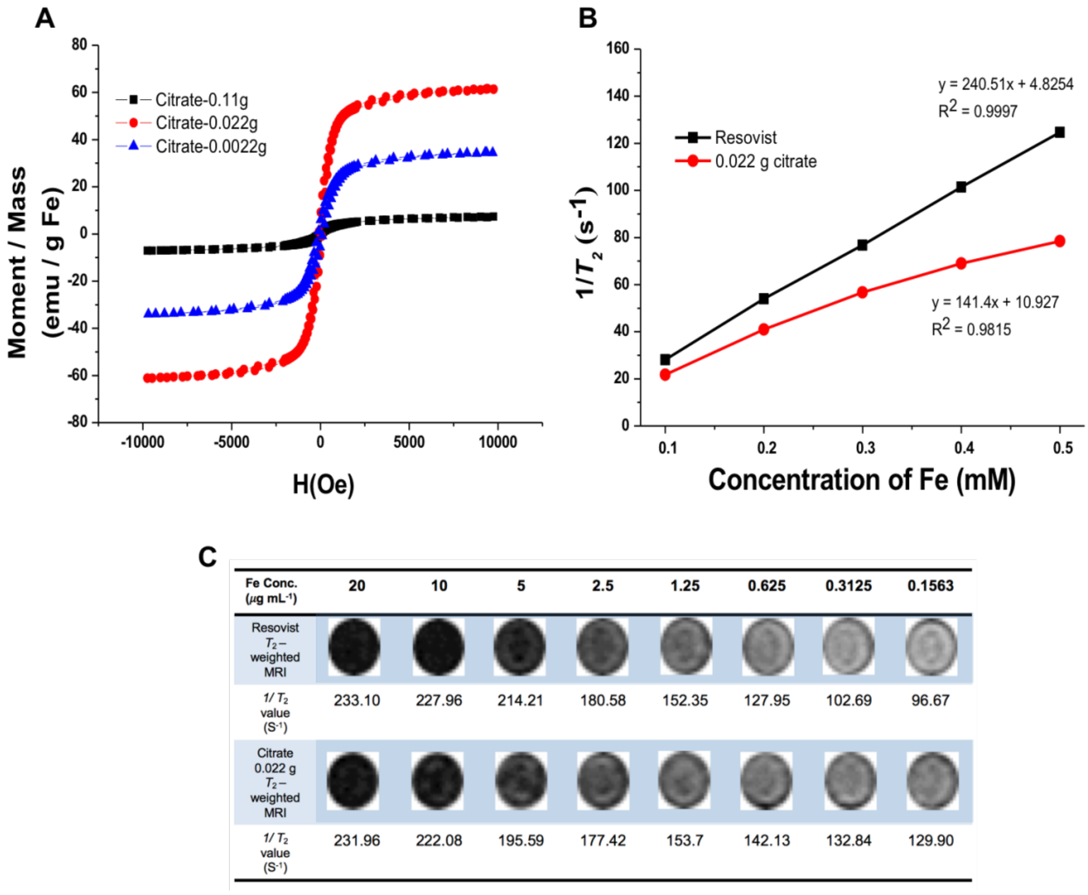

Figure S4. A) Hysteresis of NIR-IO nanocrystals synthesized using different amounts of citrate measured at room temperature and ranging from -10000 to 10000 G. B) T_2 relaxation rates as a function of the Fe concentration (mM) of Resovist® and NIR-IO nanocrystals in agar gel. C) T_2-weighted MR images of NIR-IO nanocrystals and Resovist®.

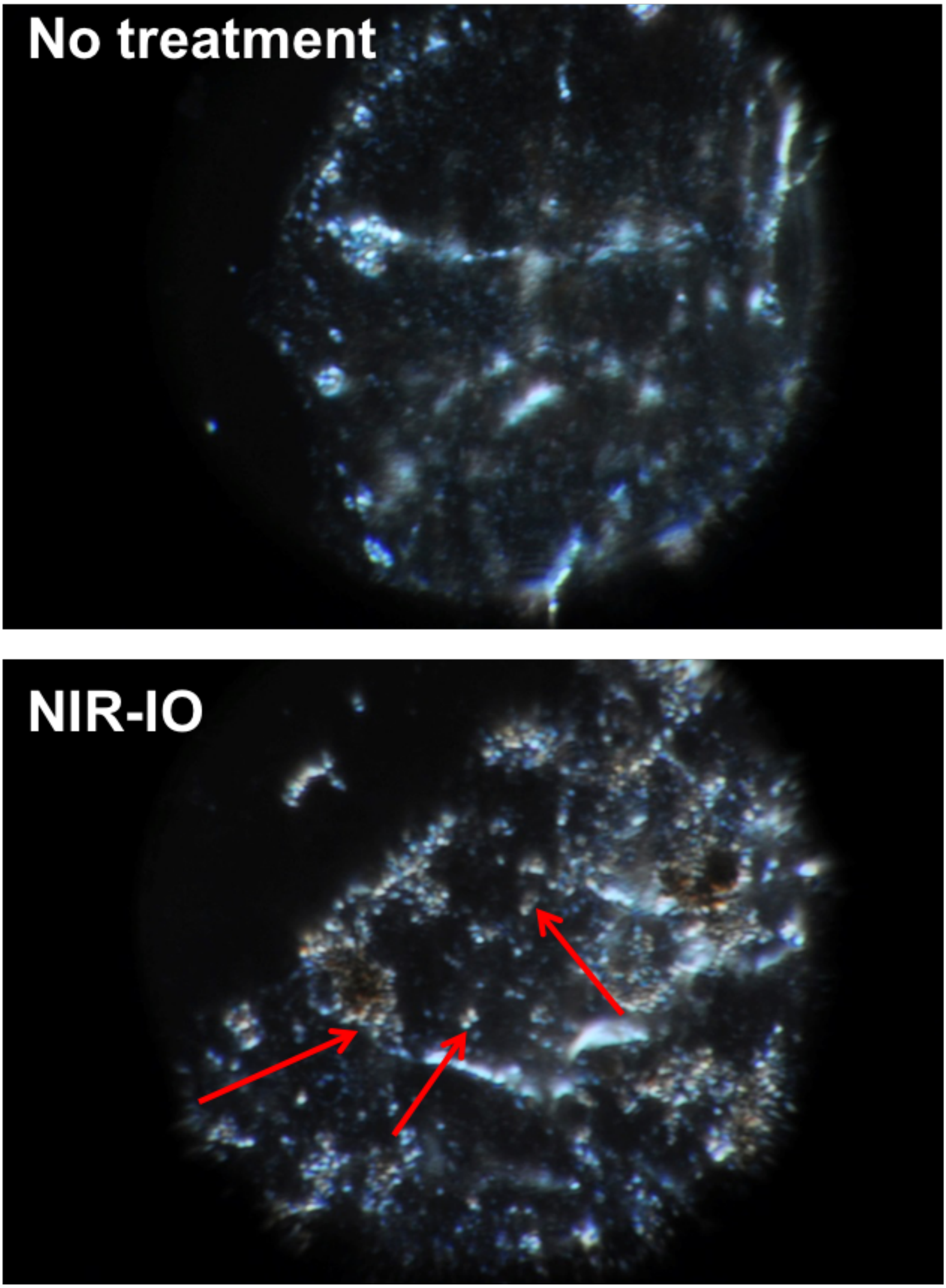

Figure S5. Dark-field reflectance images of HT-29 cancer cells treated with NIR-IO nanocrystals. The red arrows indicate nanocrystals that were internalized within the cell.

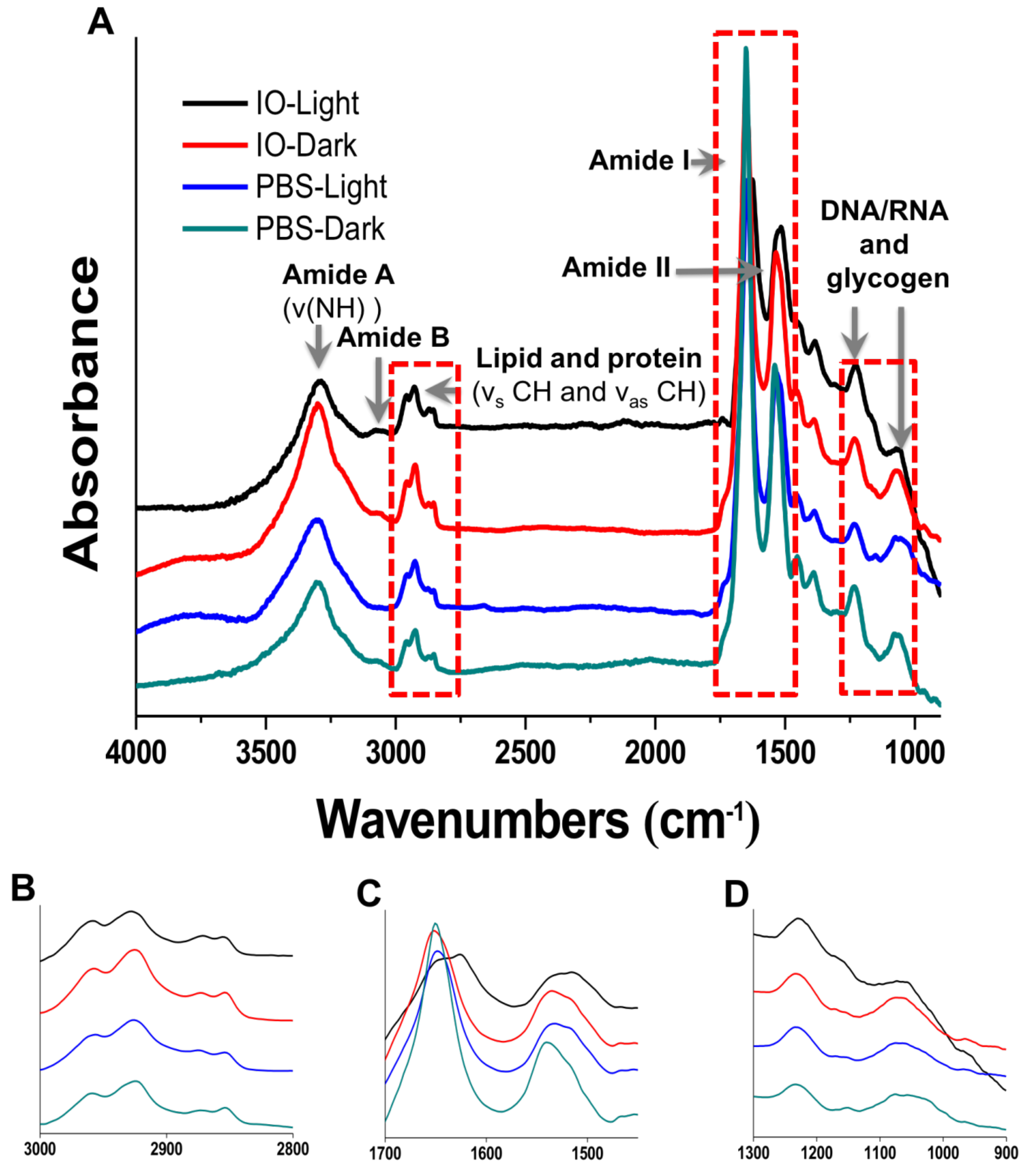

Figure S6. A) FTIR spectra of HT-29 cancer cells treated with PBS and NIR-IO nanocrystals collected by SR-IMS. The Fe concentration was 140 µg mL^{-1}. In b-d, the FTIR spectra of the treated HT-29 cancer cells were magnified to show specific ranges at wavenumbers (cm^{-1}) of B) 3000-2800, C) 1700-1400 and D) 1300-900.

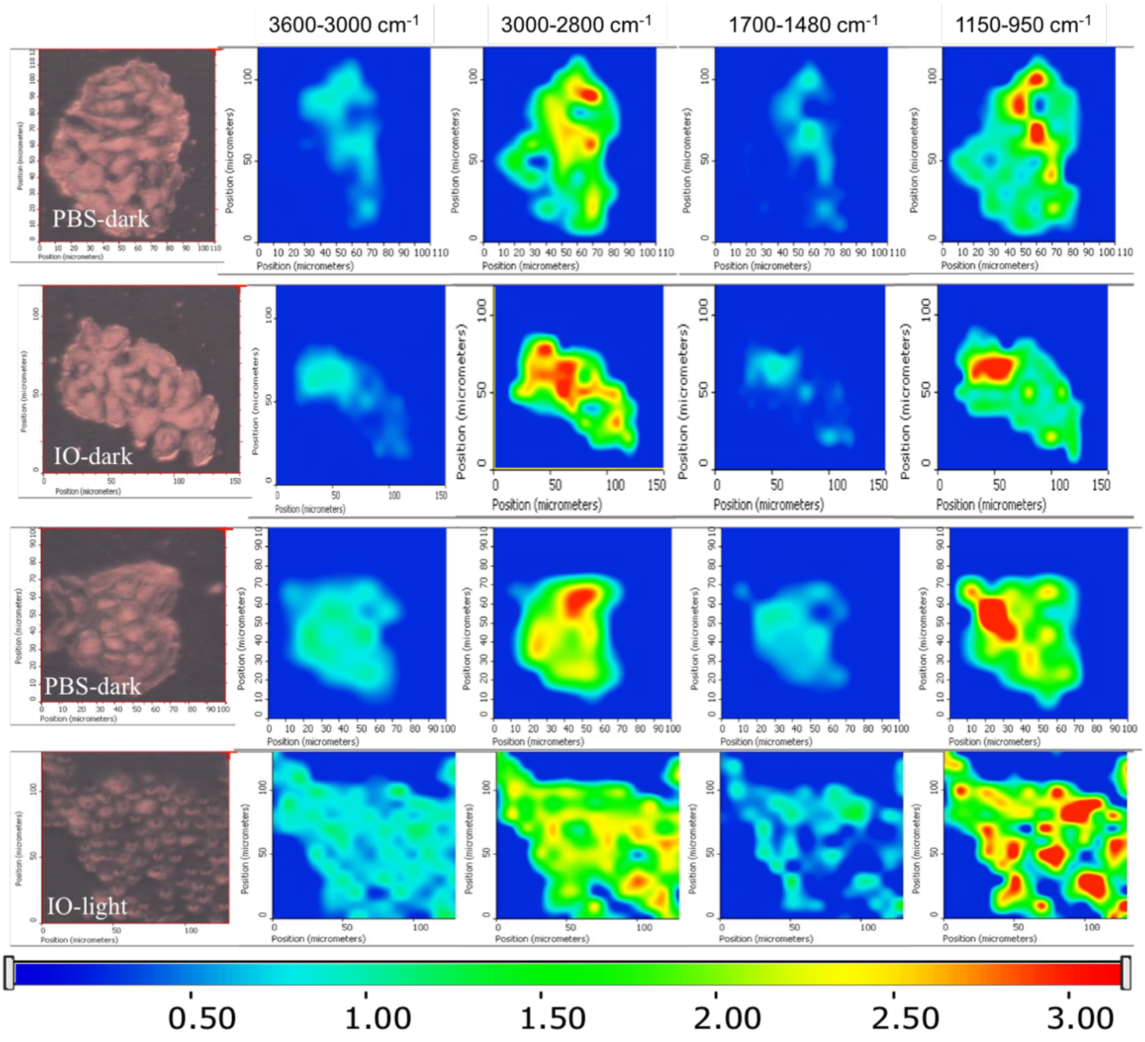

Figure S7. FTIR images of HT-29 cells incubated with PBS and NIR-IO nanocrystals. The Fe concentration was 140 μg mL^{-1}.

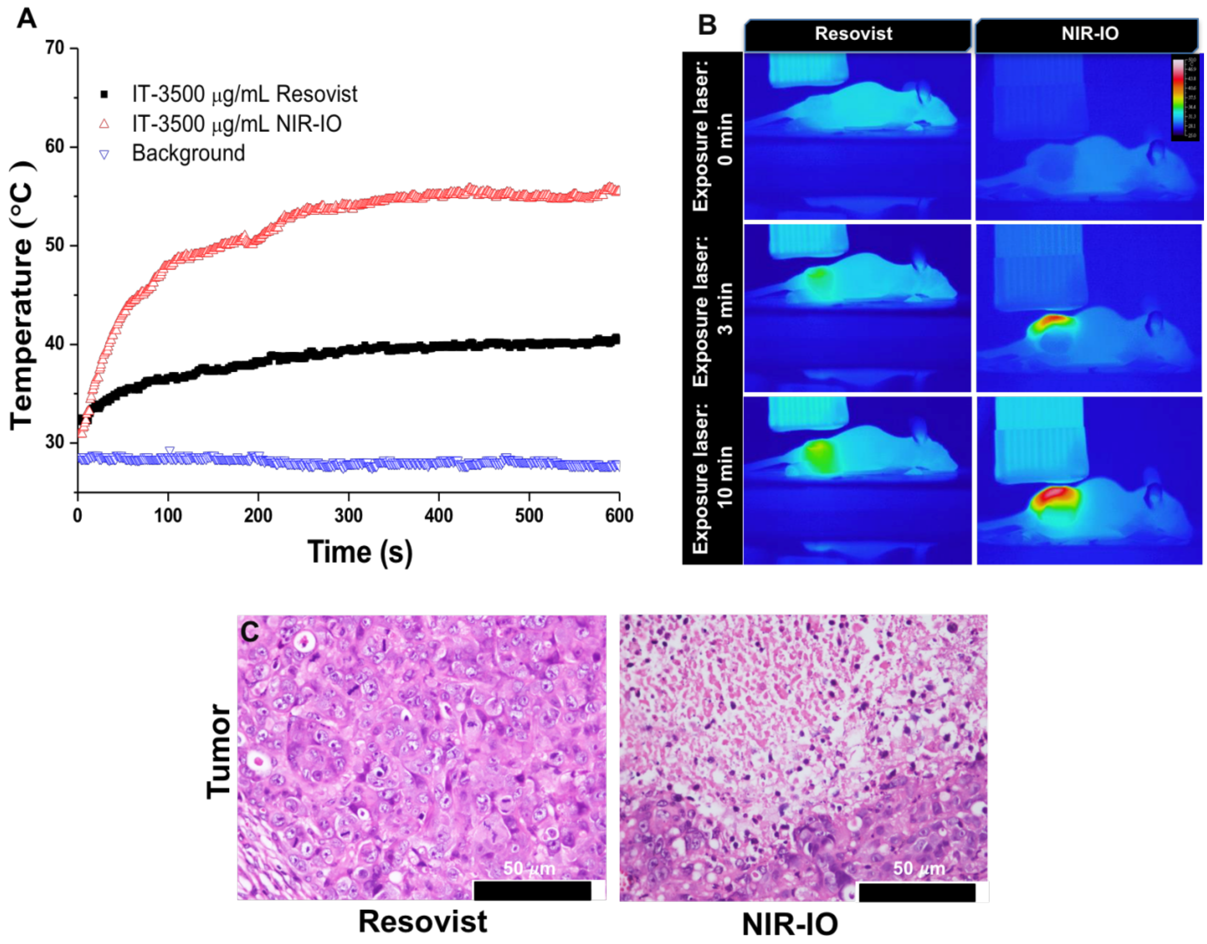

Figure S8. A) Heating curves, B) thermographic images and C) H&E-stained images of mice bearing HT-29 tumors after intratumoral injection of Resovist® or NIR-IO nanocrystals (14 mg kg^{-1}) and 10-min exposure to 808-nm laser irradiation (1.5 W cm^{-2}).

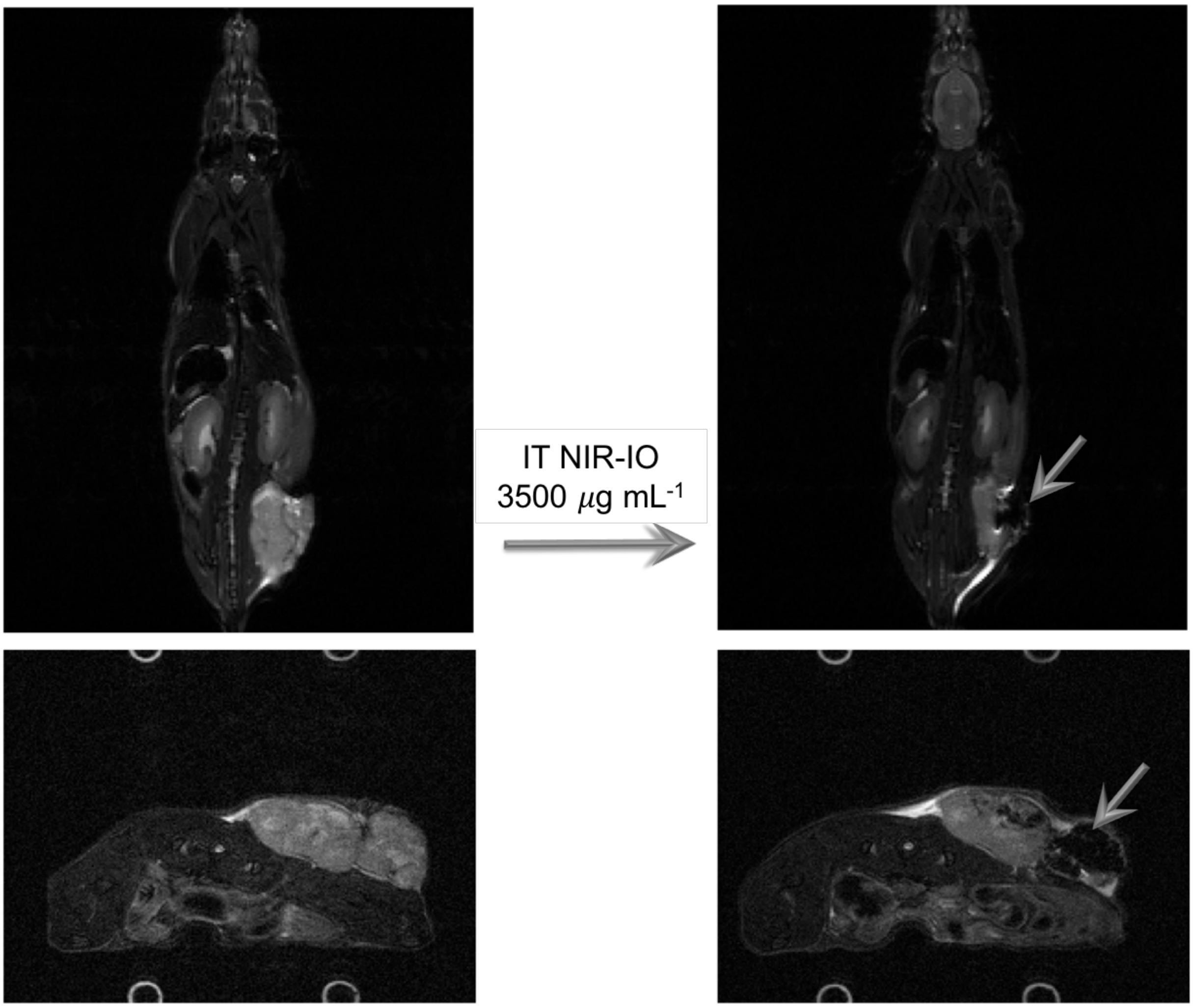

Figure S9. MR images of mice before and after IT injection of NIR-IO nanocrystals. The gray arrow indicates the site of injection of NIR-IO nanocrystals.

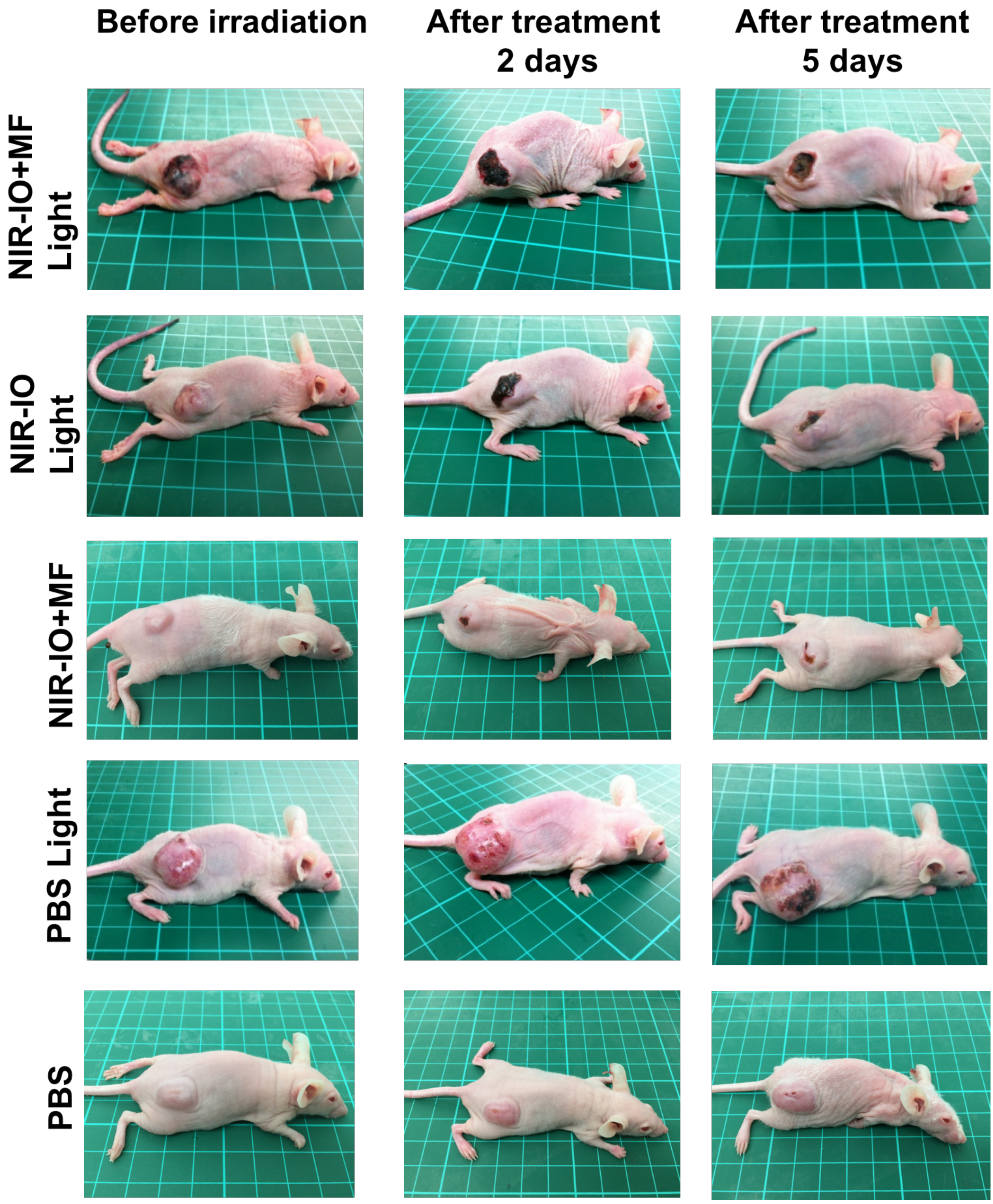

Figure S10. Optical images showing the effects of treatment with PBS, NIR-IO nanocrystals, or NIR-IO nanocrystals + MF targeting via 808-nm laser irradiation.

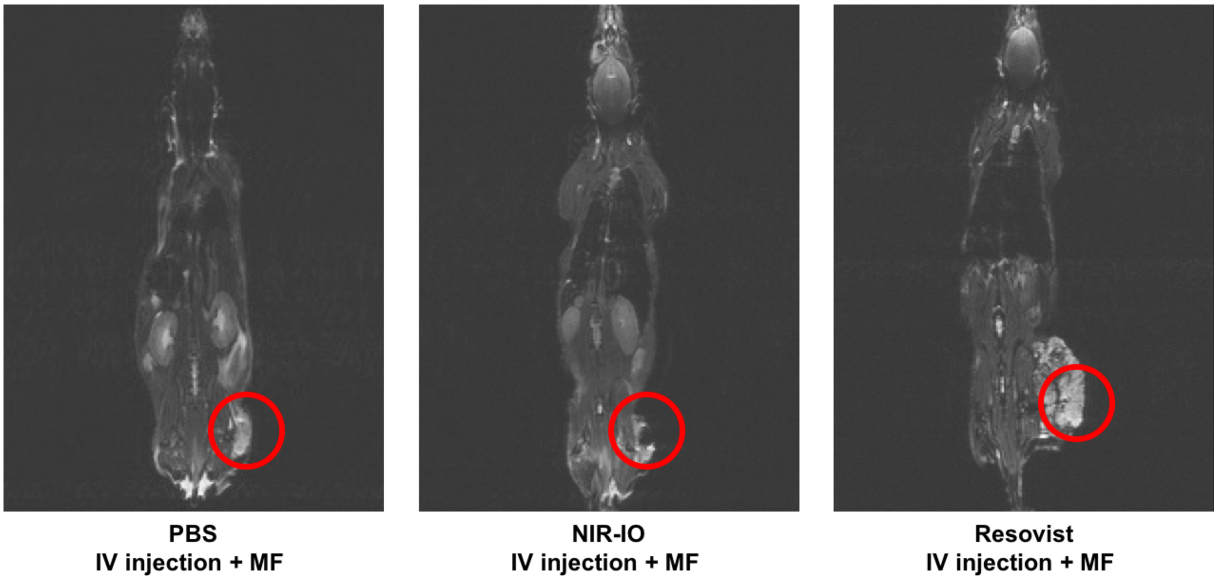

Figure S11. MR images of mice before and after IV injection + MF targeting of NIR-IO nanocrystals. The red circles indicates the site of tumor and MF targeting position.

.

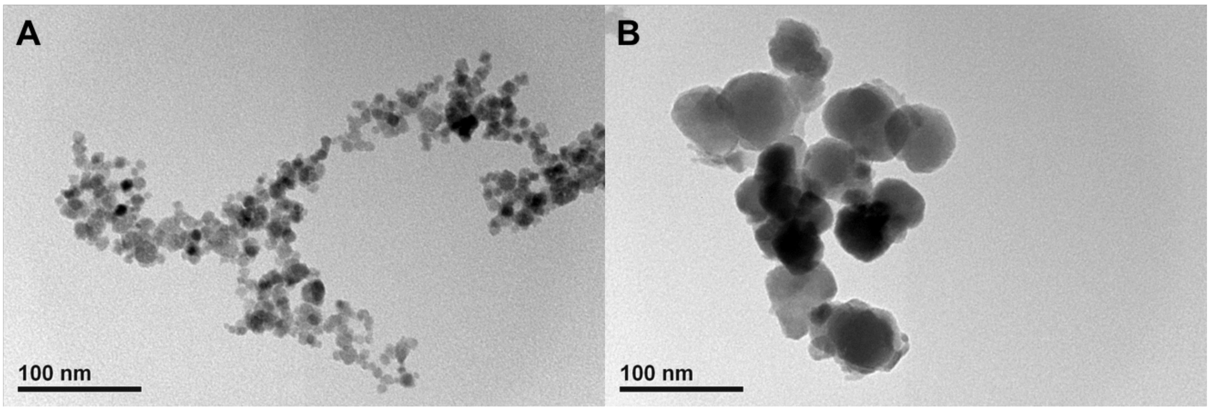

Figure S12. TEM images of NIR-IO nanocrystals synthesized with (A) 0.066 g of DPc or (B) 0.0099 g of DPc as a replacement for citrate.
